# Impact of the Cardio-Meds Mobile App on Heart Failure Knowledge and Medication Adherence: Pilot Randomized Controlled Trial

**DOI:** 10.2196/83022

**Published:** 2026-02-23

**Authors:** Victor Buswell, Emmanuelle Massie, Elena Tessitore, Lisa Simioni, Guillaume Guebey, Hamdi Hagberg, Aurélie Schneider-Paccot, Samaksha Pant, Katherine Blondon, Liliane Gschwind, Frederic Ehrler, Philippe Meyer

**Affiliations:** 1 Faculty of Medicine, University of Geneva, Geneva, Switzerland; 2 Heart Failure and Cardiovascular Rehabilitation Unit, Cardiology Service, Geneva University Hospitals, Rue Gabrielle Perret-Gentil 4, Geneva, 1205, Switzerland, (+41) 22 372 13 13, (+41) 22 372 37 45; 3 Computer Science Department, University Hospital of Geneva, Geneva, Switzerland; 4 Cardiology Division, Hôpital de la Tour, Meyrin, Switzerland; 5 Medical Directorate, University Hospital of Geneva, Geneva, Switzerland; 6 Pharmacy Department, University Hospital of Geneva, Geneva, Switzerland

**Keywords:** heart failure, mHealth, medication adherence, patient education, digital health intervention, randomized controlled trial, mobile health

## Abstract

**Background:**

Heart failure (HF) is a prevalent chronic condition for which optimal management depends not only on guideline-directed medical therapy but also on patients’ understanding of their disease, recognition of warning signs, and sustained medication adherence, which remains challenging in routine care. Mobile health interventions may support therapeutic education and self-management; however, many available apps lack validated content and local relevance. Cardio-Meds is a mobile app developed at Geneva University Hospitals to support HF self-management through structured educational content, interactive quizzes, medication lists with reminders, and tools for monitoring weight and vital signs.

**Objective:**

This study aims to evaluate the impact of a 30-day Cardio-Meds intervention on HF knowledge and medication adherence in patients with HF with reduced or mildly reduced ejection fraction.

**Methods:**

We conducted a single-center, pilot randomized controlled trial in patients followed at the outpatient HF clinic or enrolled in cardiac rehabilitation at Geneva University Hospitals in 2024. Eligible participants had HF with a left ventricular ejection fraction less than 50%, were receiving HF-specific pharmacotherapy, speak French, and owned a smartphone. Participants were recruited by phone and randomized to Cardio-Meds use for 30 days, a self-guided intervention with a single standardized technical support call. Outcomes were self-assessed using standardized questionnaires: HF knowledge and self-management using the Dutch Heart Failure Knowledge Scale (DHFKS; score range 0‐15); medication adherence using the Basel Assessment of Adherence to Immunosuppressive Medication Scale, covering initiation, implementation, and persistence; and usability in the intervention group using the System Usability Scale (score range 0‐100). Between-group differences in DHFKS scores were analyzed using analysis of covariance adjusted for baseline values.

**Results:**

A total of 49 participants were included (25 intervention, 24 control); 78% (n=38) were male, and the mean age was 62 (SD 11.4) years. In the intervention group, median app usage was 123 (IQR 74‐273) minutes, with a median of 43 (IQR 19‐85) logins. Mean baseline DHFKS scores were similar between groups (intervention 11.1, SD 2.4 vs control 10.5, SD 2.9). At 30 days, mean scores increased significantly in the intervention group (12.4, SD 2.4; mean change +1.3; *P*<.001) and remained stable in the control group (10.4, SD 3; mean change –0.1; *P*=.82), with a significant adjusted between-group difference of +1.3 points (*P*<.001). No significant between-group differences were observed for medication adherence. Usability was high, with a mean score of 84.3 (SD 15), and 64% (16/25) of intervention participants reported that they would continue using the app.

**Conclusions:**

In a stable ambulatory HF population, the Cardio-Meds intervention demonstrated short-term improvement in HF knowledge, while no effect was observed on medication adherence within the 30-day follow-up period. The app showed high usability and acceptability. Larger multicenter studies with longer follow-up are needed to assess clinical impact.

## Introduction

Heart failure (HF) is a chronic disease affecting more than 64 million people worldwide [1], including approximately 200,000 in Switzerland [2]. Its prevalence is increasing due to aging populations and improved survival. Despite advances in therapy, HF remains a leading cause of morbidity, mortality, and health care utilization. In the European Society of Cardiology-HF Long-Term Registry, 1-year all-cause mortality after hospitalization for acute HF was 23.6%, with a combined incidence of death or HF-related readmission reaching 36% [3]. Readmissions contribute significantly to the clinical and economic burden of HF. In Europe and North America, HF accounts for 1% to 2% of total health care expenditures, largely due to hospitalizations [4,5]. Effective outpatient strategies are therefore crucial. Guideline-directed therapy for patients with reduced or mildly reduced ejection fraction (HFrEF and HFmrEF) includes 4 foundational drug classes: angiotensin receptor–neprilysin inhibitors, angiotensin receptor blockers or angiotensin-converting enzyme inhibitors, beta-blockers, mineralocorticoid receptor antagonists, and sodium-glucose cotransporter 2 inhibitors [6]. These treatments clearly improve outcomes but require a high level of adherence, which remains challenging in patients with multiple comorbidities.

Therapeutic education and structured follow-up are therefore central to improving adherence and clinical outcomes in patients with HF. Cardiac rehabilitation programs, recommended by both European and American guidelines (class I, level A), reduce readmissions and improve quality of life [7]. Broader evidence also supports educational interventions in improving self-care behaviors and adherence [8-10]. A meta-analysis by Van Spall et al [8] demonstrated reduced readmissions with nurse-led follow-up and therapeutic education, while Ruppar et al [10] found lower mortality with adherence-focused interventions. However, scalability and patient engagement remain challenges. Mobile health (mHealth) technologies offer accessible, personalized, and cost-effective support. Self-management apps provide education, monitoring, and behavioral reinforcement outside clinical settings. The 2021 European Society of Cardiology guidelines recommend self-management strategies to reduce HF-related hospitalizations and mortality [11,12].

Recent randomized controlled trials (RCTs) have evaluated multicomponent mHealth interventions integrating mobile apps and connected devices, reporting improvements in self-care behaviors and symptom outcomes in HF populations. Kitsiou et al [13] tested the iCardia4HF program in a phase 1 RCT of combined consumer apps and feedback messages, demonstrating feasibility and preliminary efficacy on self-care measures. Moreover, the SMART-HF study employed a smartphone app with remote monitoring and feedback, showing symptomatic benefits and supporting the role of digital strategies in HF management [14]. Systematic reviews of recent RCTs also highlight the potential of digital health interventions to affect clinical outcomes and self-care, though evidence remains heterogeneous [15].

However, current HF apps often lack validated content and integration into national health care systems. Dunn Lopez et al [16] found that most apps were difficult to understand, poorly referenced, and lacked personalized feedback. Apps such as WOW ME 2000mg, WebMD, and My Cardiac Coach provide generic tools but do not account for local medications or structures [17-19]. Usability, health literacy, and personalization remain key success factors for mobile apps [20,21].

This RCT evaluates the impact of Cardio-Meds, an mHealth app developed by the Cardiology Department at Geneva University Hospitals (HUG) [22], on HF knowledge and therapeutic adherence in patients with HFrEF or HFmrEF. The study aims to determine whether a mobile intervention can enhance disease understanding and support adherence in a real-world outpatient setting.

## Methods

### Study Design

This single-center, prospective, pilot RCT was conducted at HUG. Participants were randomly assigned to either the intervention group (using the Cardio-Meds app) or the control group (usual care). A total of 50 participants were planned for inclusion, with 25 participants per group. The primary objective was to assess whether Cardio-Meds improves knowledge and self-management of HF compared with usual care. Secondary and exploratory objectives were to evaluate its impact on medication adherence and user satisfaction. The study was designed as an exploratory evaluation of short-term effects on HF knowledge, feasibility, and usability rather than as a definitive efficacy trial for medication adherence.

### Ethical Considerations

The study was submitted to the local ethics committee (Commission cantonale d’éthique de la recherche, Geneva, Switzerland; ID 2023‐01337) for review. The Commission cantonale d’éthique de la recherche reviewed the submission (ID 2023‐01337) and determined that formal approval was not required given the low-risk, educational nature of the study and the absence of clinical or safety end points. All participants provided written informed consent prior to participation. Participant privacy and confidentiality were maintained. Study data were securely stored at Geneva University Hospitals and analyzed in pseudonymized form. Participants did not receive any financial compensation for participation.

This exploratory, single-center RCT was not prospectively registered in a public trial registry. At the time of study initiation, prospective registration was not requested for this type of low-risk educational intervention. All study outcomes, analyses, and end points were predefined before data collection and are reported consistently, with no outcome switching. Future larger-scale or multicenter randomized trials evaluating Cardio-Meds will be prospectively registered in accordance with international recommendations.

### Description of the Intervention (Cardio-Meds App)

Cardio-Meds is a research version of a mHealth app developed at HUG through collaboration between cardiology, pharmacy, the medical directorate, and IT departments. A recent usability study rated it good to excellent, supporting its feasibility in the HF population [23]. The app was designed with and for patients to help individuals with HF manage their condition through educational resources, self-monitoring, and adherence support.

**Figure 1.**
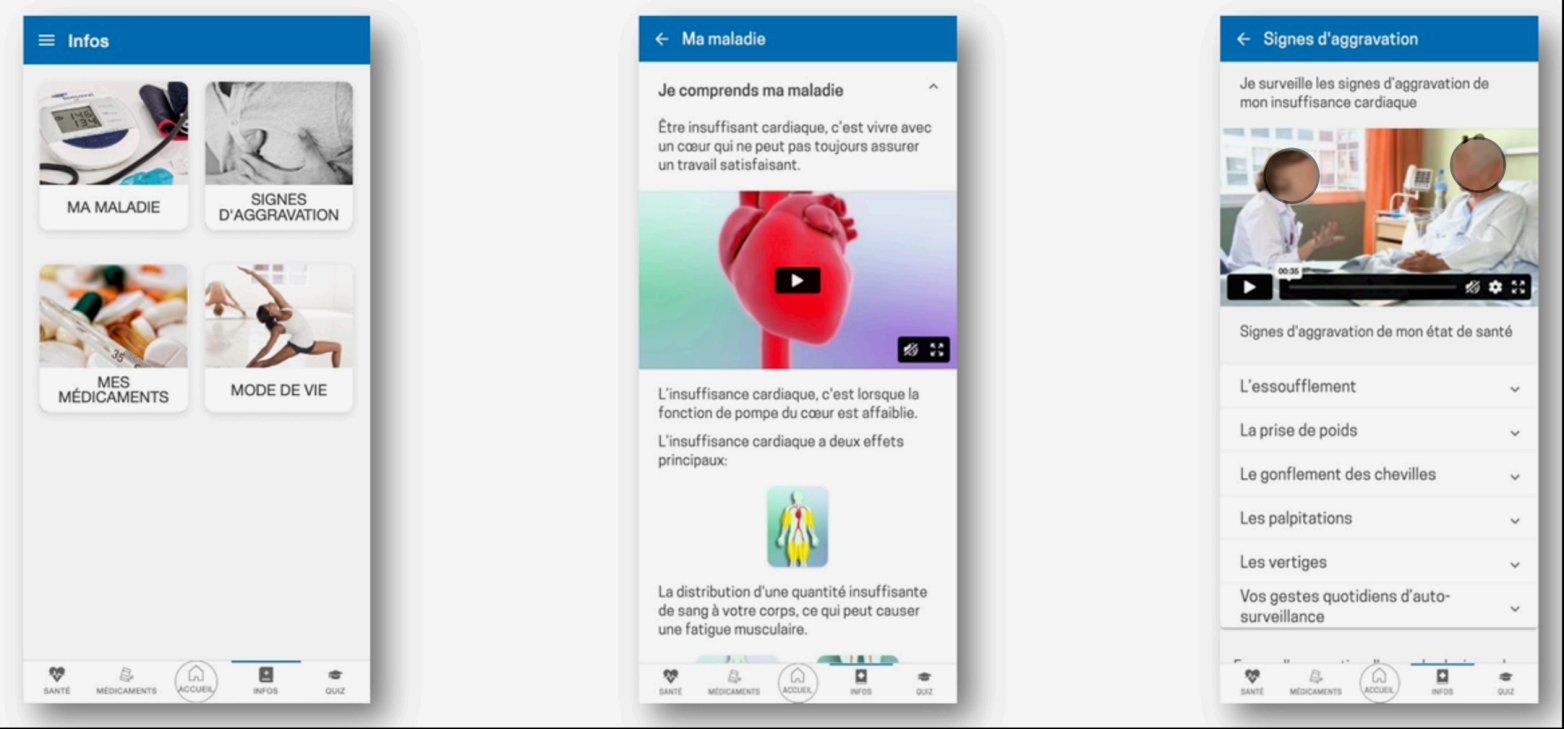
Educational information section of the Cardio-Meds mobile app for heart failure self-management. Screenshots show the structured educational content available in the Cardio-Meds app, organized into thematic modules (disease understanding, warning signs, medications, and lifestyle). The app was evaluated in a single-center pilot randomized controlled trial conducted at Geneva University Hospitals (Geneva, Switzerland) among adult outpatients with heart failure during a 30-day intervention period in 2024.

**Figure 2.**
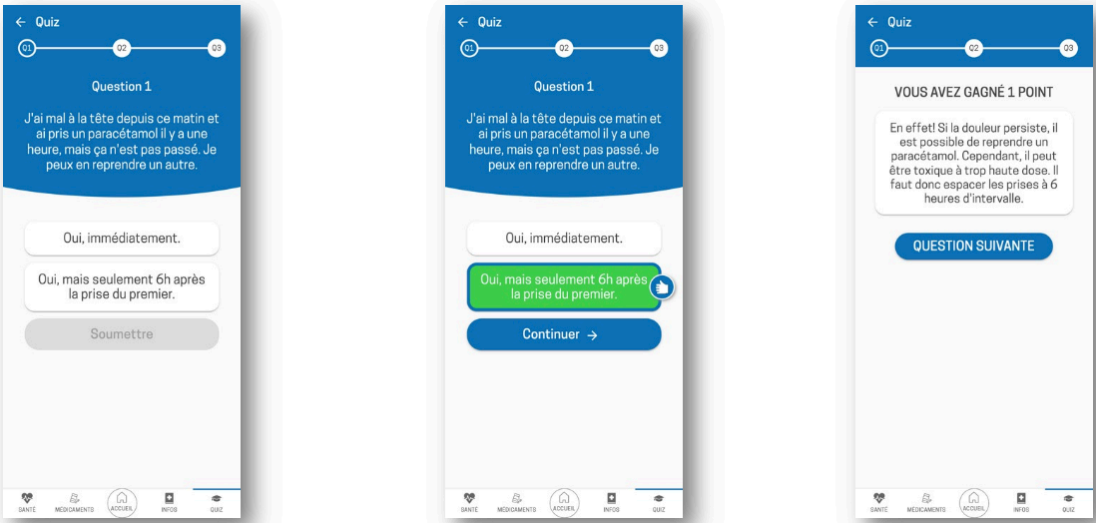
Interactive quiz module of the Cardio-Meds mobile app for heart failure self-management. Screenshots depict the quiz interface of Cardio-Meds, including multiple-choice questions and immediate feedback with brief educational explanations. The app was evaluated in a single-center pilot randomized controlled trial conducted at Geneva University Hospitals (Geneva, Switzerland) among adult outpatients with heart failure during a 30-day intervention period in 2024*.*

**Figure 3.**
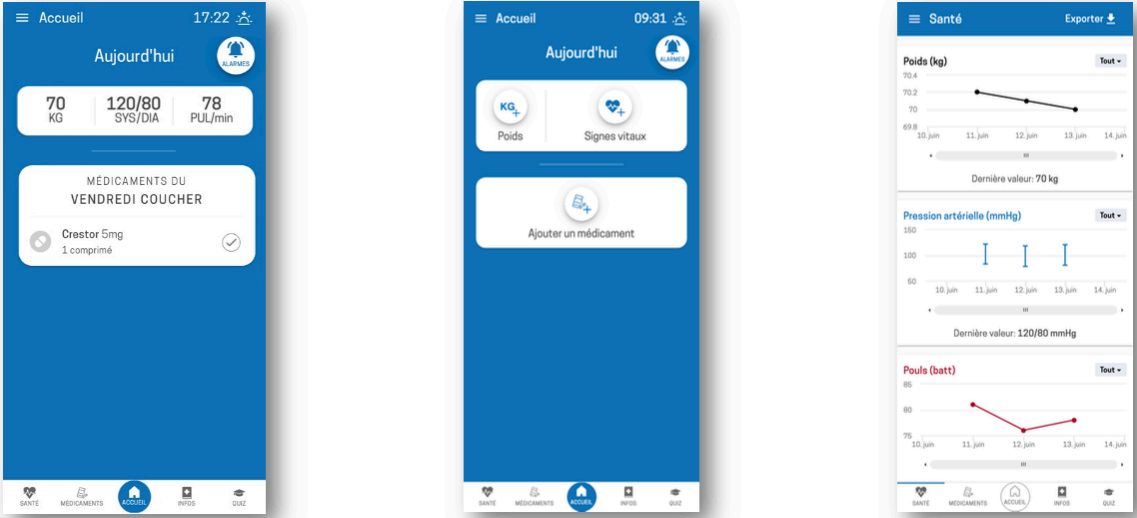
Home screen and health monitoring section of the Cardio-Meds mobile app. Screenshots illustrate the home interface and the health monitoring module of Cardio-Meds app. Shown features include daily medication overview, intake confirmation, and graphical tracking of self-reported weight, blood pressure, and heart rate. The app was evaluated in a single-center pilot randomized controlled trial conducted at Geneva University Hospitals (Geneva, Switzerland) among adult outpatients with heart failure during a 30-day intervention period in 2024. All data displayed in the app screenshots are fictitious and were entered solely for illustrative purposes.

**Figure 4.**
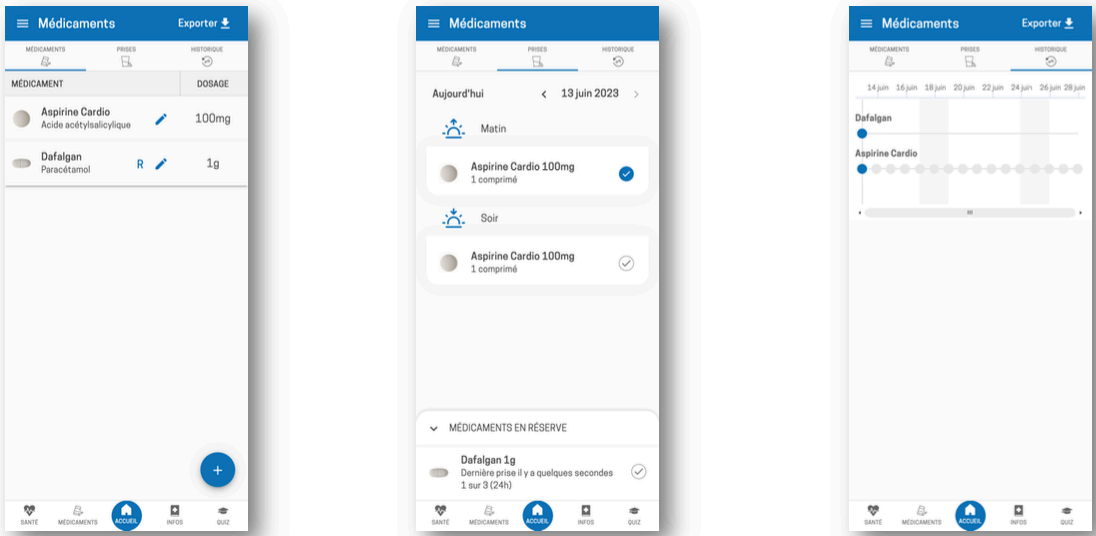
Medication management section of the Cardio-Meds mobile app (treatment list, intake confirmation, and history). Screenshots illustrate the medication section of Cardio-Meds, including the individualized treatment list, daily intake confirmation, and medication history. The app was evaluated in a single-center pilot randomized controlled trial conducted at Geneva University Hospitals (Geneva, Switzerland) among adult outpatients with heart failure during a 30-day intervention period in 2024. All data displayed in the app screenshots are fictitious and were entered solely for illustrative purposes.

**Figure 5.**
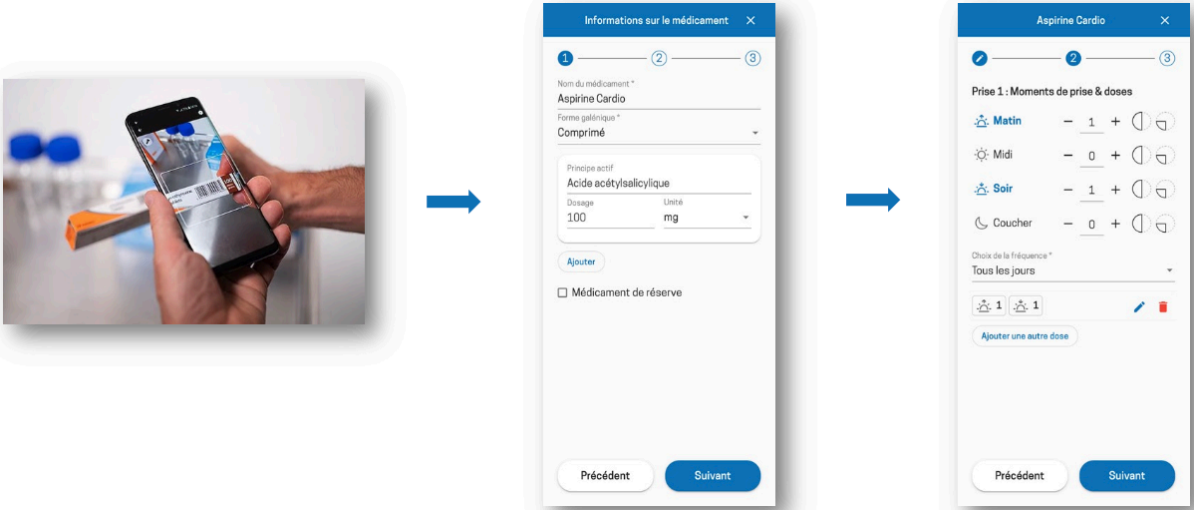
Procedure for entering a medication into the Cardio-Meds mobile app. Screenshots show the step-by-step process for adding a medication in Cardio-Meds, including barcode scanning linked to the Swiss medication database, dosage specification, and intake scheduling. The app was evaluated in a single-center pilot randomized controlled trial conducted at Geneva University Hospitals (Geneva, Switzerland) among adult outpatients with heart failure during a 30-day intervention period in 2024.

**Figure 6.**
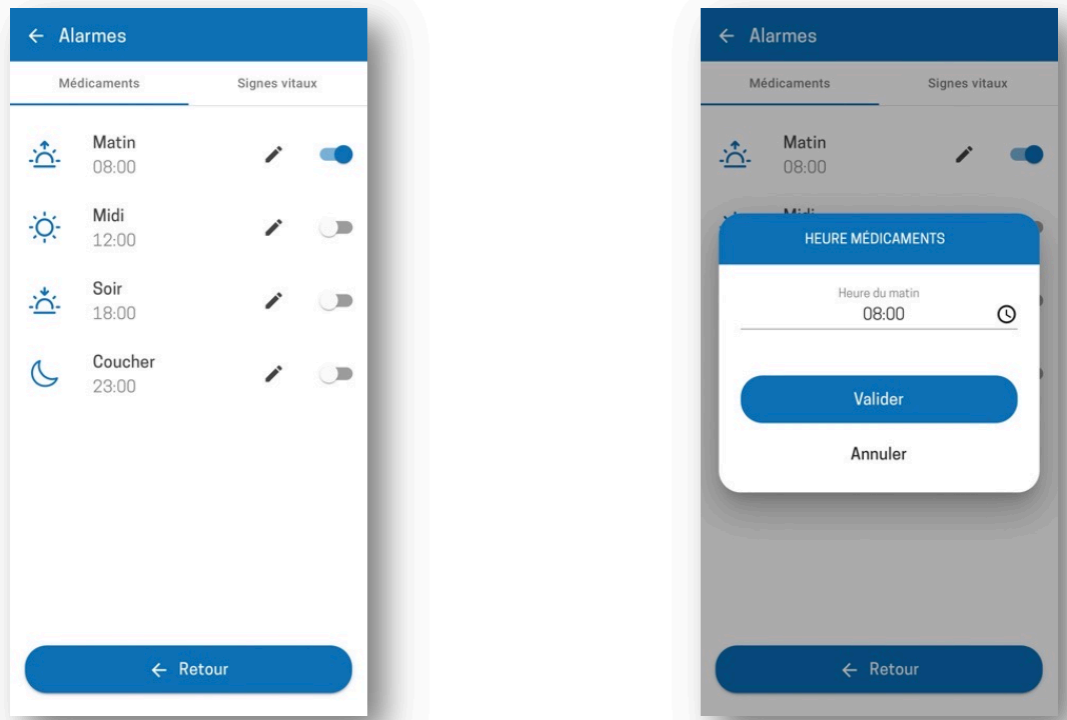
Procedure for setting medication reminder alarms in the Cardio-Meds mobile app. Screenshots illustrate the configuration of medication reminder alarms, including timing and activation of notifications. These reminders are optional. The app was evaluated in a single-center pilot randomized controlled trial conducted at Geneva University Hospitals (Geneva, Switzerland) among adult outpatients with heart failure during a 30-day intervention period in 2024*.*

Key features include the following:

- Educational content and daily quizzes on HF, its treatment, and lifestyle. The content is organized into 4 chapters (my condition, warning signs, my medication, and lifestyle, Figure 1). Quizzes provide immediate feedback and brief explanations (Figure 2).
- Tools to track weight, blood pressure, and heart rate, enabling early detection of decompensation. Graphs can be generated and used during consultations (Figure 3).
- A treatment plan, easily accessible on the user’s phone, allows patients to enter their medication regimen (Figure 4). Medications can be added by scanning the barcode, enabling the app to identify the drug via access to the Swiss medication database. The user can then specify the dosage and timing for each medication intake (Figure 5).
- Intake confirmation and optional reminders to support adherence (Figures3, 4,  6).

The Cardio-Meds app enables patients to manage health data, access structured learning, and share information with health care providers. It was designed to ensure ease of use and fulfill legal and best practice requirements for data protection and confidentiality.

### Study Population and Recruitment

Participants were recruited from the HF clinic and cardiac rehabilitation programs at HUG from March to November 2024. Two cardiologists and 2 specialized HF nurses preidentified eligible participants, who were contacted by a medical student who explained the study and verified smartphone ownership. Participation was voluntary, with the option to withdraw at any time without affecting medical care.

Participants were eligible for inclusion if they met the following criteria:

- Aged 18 years or older
- Diagnosed with HF with reduced (≤40%, or HFrEF) or mildly reduced (41%‐49% or HFmrEF) left ventricular ejection fraction
- Currently receiving HF-specific medications
- No cognitive impairment and fully capable of decision-making
- Capability to read, understand, and communicate in French
- Ownership of a smartphone

Participants were excluded if they met any of the following conditions:

- Inability or unwillingness to comply with study procedures
- Status as an asylum seeker, individuals experiencing homelessness, or detainees

### Study Procedures

**Figure 7.**
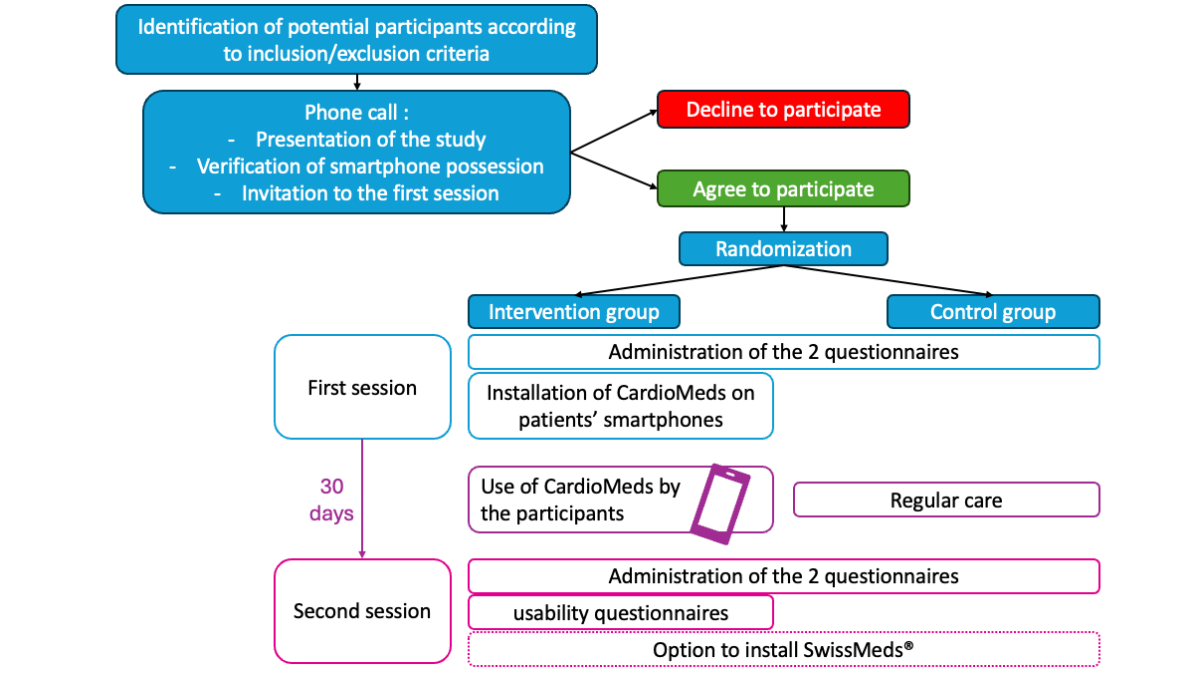
Flowchart of the study design and procedures of a pilot randomized controlled trial evaluating the Cardio-Meds mobile app. The figure outlines participant recruitment, randomization, intervention, and follow-up procedures in a single-center pilot randomized controlled trial conducted at Geneva University Hospitals (Geneva, Switzerland) between March and November 2024. Adult patients with heart failure were randomized to either a 30-day Cardio-Meds intervention plus usual care or to usual care alone. Heart failure knowledge and medication adherence were assessed at baseline and at 30 days using the Dutch Heart Failure Knowledge Scale (DHFKS) and the Basel Assessment of Adherence to Immunosuppressive Medication Scale (BAASIS*)*.

After providing oral consent and before the first session, participants were randomized using a computer-generated random allocation sequence with a 1:1 ratio between the intervention and control groups, without stratification or blocking. The allocation sequence was generated by a member of the research team not involved in participant recruitment or outcome assessment. Randomization was conducted prior to the first study session for logistical reasons related to group-based study organization. Participants were enrolled by study investigators, and baseline questionnaires were administered before any information regarding group allocation was disclosed (Figure 7). Neither participants nor study personnel responsible for distributing and collecting the questionnaires were aware of group assignment at the time of baseline data collection. Group allocation was revealed only after completion of baseline assessments, at which point participants in the intervention group received instructions for app installation. Given the nature of the digital intervention, blinding of participants and study staff after allocation was not feasible. Outcome assessment relied on self-administered questionnaires, and data analysis was conducted on deidentified datasets by analysts not involved in intervention delivery.

During session 1, after written informed consent, participants completed 2 baseline assessments:

- The Dutch Heart Failure Knowledge Scale (DHFKS) assessing HF-related knowledge and self-management
- The Basel Assessment of Adherence to Immunosuppressive Medication Scale (BAASIS) evaluating medication adherence

Intervention group participants installed the Cardio-Meds app on their smartphones with guidance on usage and for medication entry. A brief standardized mid-study support call was conducted solely to identify and resolve potential technical issues related to app installation or functionality. No medical advice, educational reinforcement, or adherence-related counseling was provided during this call. After 30 days, all participants returned for a second session, completing the DHFKS and BAASIS again. Intervention participants also completed a usability questionnaire and had the possibility to give nondirected feedback on the app. At the end of the study, both groups could install SwissMeds, the publicly released version of the app, soon to include modules for other chronic conditions.

Most participants attended group sessions (5‐12 participants) held in March, May, and November 2024. Remaining participants were seen individually during the week following each of these sessions.

The following anonymized usage data were collected from the app:

- Total time spent on the app
- Number of logins
- Time spent on the information section
- Number of weight, blood pressure, and heart rate entries over the 30-day follow-up period
- Percentage of correct answers to the quizzes

Clinical and personal data were collected from the electronic medical record at HUG, anonymized, and securely stored on HUG servers for 10 years in compliance with institutional policy.

### Outcome Measures

#### Primary Outcome

HF knowledge and self-management were assessed using the DHFKS, a validated 15-item questionnaire covering general knowledge, dietary recommendations, sodium and fluid restrictions, and recognition of alarm symptoms. This tool was selected for its validity and sensitivity in evaluating the effect of therapeutic education in patients with HF. The questionnaire was translated into French using neural machine translation (DeepL) and reviewed for clinical accuracy and clarity by an experienced HF specialist (PM). No formal forward-backward translation or psychometric validation of the French version was performed, and the scale was therefore used as an exploratory measure to assess within-group changes in knowledge rather than as a fully validated French-language instrument. Scores range from 0 to 15, with higher scores indicating better knowledge. Based on pragmatic and clinical considerations, an absolute improvement of 2 points on the DHFKS was prespecified as a meaningful change for sample size estimation. This threshold was used as an assumption for planning purposes rather than as a validated minimal clinically important difference (Multimedia Appendix 1).

#### Secondary Outcome

Medication adherence was assessed using the BAASIS, a structured self-report questionnaire originally validated in transplant populations. In the absence of an HF-specific validated adherence questionnaire at the time of study design, the BAASIS was used as an exploratory tool to capture core adherence behaviors (initiation, implementation, and persistence) relevant to chronic disease management. BAASIS covers three dimensions of adherence behavior:

- Initiation (starting newly prescribed medications during the reference period)
- Implementation (missed doses, wrong dosage, or timing)
- Persistence (discontinuation without medical advice)

Responses are binary (“yes”/“no”), with frequency details for selected items. For the “Initiation” domain, analyses were restricted to participants who had a newly prescribed medication during the respective reference period, as specified by the BAASIS instrument; therefore, denominators vary across time points and do not reflect missing data or participant exclusion. A validated French version was used (Multimedia Appendix 2).

#### Exploratory Outcome

Usability of the app was evaluated in the intervention group using the System Usability Scale (SUS), a 10-item questionnaire with responses on a 5-point Likert scale. The final score ranges from 0 to 100, with higher scores reflecting better usability (Multimedia Appendix 3).

### Statistical Analysis

Descriptive statistics were used to summarize baseline characteristics and outcomes. Continuous variables are presented as mean (SD) and were compared between groups using Student *t* test when appropriate. Categorical variables are presented as frequencies and percentages and were compared using chi-square or Fisher exact tests, depending on expected cell counts.

The primary outcome, change in HF knowledge, was analyzed using an ANCOVA, with the postintervention DHFKS score as the dependent variable, study group as the independent variable, and baseline DHFKS score as a covariate. No post hoc adjustment was performed for other baseline variables, given the limited sample size and exploratory nature of this pilot study.

### Analysis Population

All outcome analyses were conducted according to randomized group assignment. Primary and secondary outcome analyses included all participants who completed both baseline and follow-up assessments (completers-only analysis). Participants with missing follow-up data were not included in the corresponding outcome analyses. No participants were excluded post randomization due to intervention-related reasons; analyses were restricted to participants with available baseline and follow-up data for the corresponding outcomes. Secondary outcomes related to medication adherence, assessed using the BAASIS questionnaire (initiation, implementation, and persistence domains), were analyzed descriptively and compared between groups using chi-square or Fisher exact tests, as appropriate. Usability outcomes measured by the SUS and app usage metrics were analyzed descriptively.

The sample size was estimated to inform feasibility rather than to power a definitive efficacy trial. Based on a mean control group DHFKS score of 10.9 (SD 2.3), an anticipated absolute between-group difference of 2 points, a power of 80%, and a 2-sided significance level (α) of .05, an initial sample size of approximately 40‐45 participants was estimated. Allowing for an anticipated dropout rate of 25% to 30%, a target sample size of 50 participants (25 per group) was considered feasible and appropriate for this pilot exploratory RCT. The study was powered for the primary knowledge outcome only and was not designed to detect differences in secondary outcomes such as medication adherence, particularly in a population with high baseline adherence.

All analyses were performed using R software (version 4.4.2; R Foundation for Statistical Computing). Statistical significance was defined as a 2-sided *P*<.05.

## Results

### Participants Characteristics

**Figure 8.**
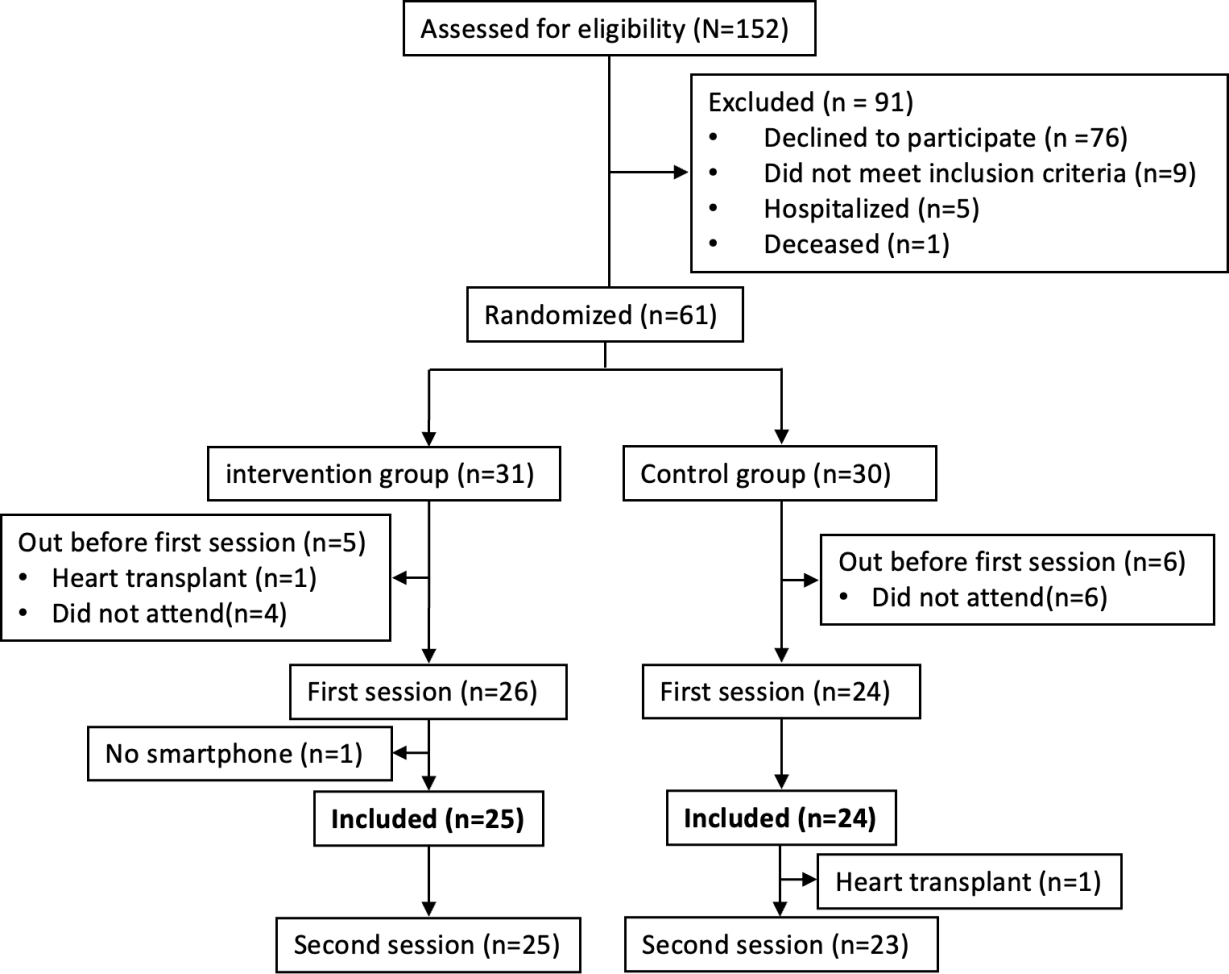
Flowchart of patient recruitment, randomization, and follow-up. The flow diagram shows screening, inclusion, randomization, and follow-up of adult outpatients with heart failure recruited at Geneva University Hospitals (Geneva, Switzerland) between March and November 2024 for a pilot randomized controlled trial of the Cardio-Meds mobile app.

A total of 152 patients with stable HFrEF or HFmrEF followed in the outpatient clinic or undergoing rehabilitation at HUG were preselected by nurses (Figure 8). After the initial phone contact, 61 patients met the eligibility criteria and agreed to participate, while 91 were excluded: 76 declined participation, 9 did not meet the inclusion criteria, 5 were hospitalized, and 1 had died. Among the 61 participants, 31 were randomly assigned to the intervention group and 30 to the control group. Before the first session, 11 participants dropped out: 10 did not attend the scheduled session, and 1 participant in the intervention group underwent heart transplantation. Ultimately, 50 participants attended the first session, and 49 were included, as 1 participant did not own a smartphone. During follow-up, 1 participant in the control group underwent heart transplantation and was therefore unable to attend the second session, and 2 participants did not complete the BAASIS questionnaire at follow-up.

This pilot study included a total of 49 adult participants with HF, of whom 78% (38/49) were male, with a mean age of 62 (SD 11.4) years. Most participants were French- or Italian-speaking and demonstrated a good level of comprehension of French. Both ischemic and nonischemic etiologies of HF were represented, with a mean left ventricular ejection fraction of 37.2% (SD 12). Participants were generally polymorbid, with common comorbidities including diabetes, hypertension, and atherosclerotic cardiovascular disease. All participants were receiving guideline-directed medical therapy, including angiotensin-converting enzyme inhibitor/angiotensin receptor blocker/angiotensin receptor–neprilysin inhibitors, beta-blockers, mineralocorticoid receptor antagonists, and sodium-glucose cotransporter-2 inhibitors. The average number of daily medications and intakes was high (more than 8 medications per day), reflecting the complexity of treatment in this population. Overall, the cohort was representative of a typical population of individuals with HF managed in an outpatient and rehabilitation setting (Table 1).

**Table 1.** Baseline characteristics of participants included in a pilot randomized controlled trial of a mobile health intervention for heart failure (N=49).^a^

Characteristic	Intervention group (n=25)^b^	Control group (n=24)^b^	*P* value^c^
Gender, n (%)			.67
Female	5 (20)	6 (25)	
Male	20 (80)	18 (75)	
Mean age (y), mean (SD)	59.2 (10.3)	65.2 (11.9)	.06
HF^d^ etiology, n (%)			.98
Ischemic	10 (40)	9 (37.5)	
Arrhythmic	4 (16)	5 (20.8)	
Valvular	3 (12)	2 (8.3)	
Toxic	6 (24)	3 (12.5)	
Genetic	6 (24%)	5 (20.8)	
Hypertensive	5 (20)	4 (16.7)	
Infiltrative	3 (12)	3 (12.5)	
Idiopathic	4 (16)	3 (12.5)	
Other	1 (4)	0 (0)	
Time since diagnosis (mo), mean (SD)	55 (85.6)	66.3 (87.6)	.65
HF medication, n (%)			>.99
ACE^e^/ARB^f^/ARNI^g^	23 (92)	22 (91.7)	
BB^h^	21 (84)	21 (87.5)	
MRA^i^	16 (64)	17 (70.8)	
SGLT2^j^	23 (92)	21 (87.5)	
Loop diuretic	13 (52)	13 (54.2)	
Number of medications, mean (SD)	8.5 (3.6)	8.9 (3.1)	.65
Daily intakes, mean (SD)	9.6 (3.8)	10 (4.3)	.76
Comorbidities, n (%)			.13
AF^k^	8 (32)	8 (33.3)	
COPD^l^	0 (0)	1 (4.2)	
PAD^m^/Stroke	8 (32)	2 (8.3)	
Diabetes	3 (12)	8 (33.3)	
Hypertension	13 (52)	10 (41.7)	
LVEF^n^, mean (SD)	38 (12)	37 (12)	.69
NYHA^o^, mean (SD)	1.8 (0.9)	1.8 (1.1)	.75
Employment status, n (%)			.56
Full-time	13 (52)	8 (33.3)	
Part-time	3 (12)	3 (12.5)	
Unemployed or disability benefits	2 (8)	2 (8.3)	
Retired	7 (28)	11 (45.8)	
Ethnicity, n (%)			.64
Caucasian	22 (88)	20 (83.3)	
African	3 (12)	4 (16.7)	
Level of French comprehension and expression, n (%)			.61
Excellent	19 (76)	19 (79.2)	
Good	5 (20)	5 (20.8)	
Average	1 (4)	0 (0)	
Mother tongue, n (%)			.25
French	12 (48)	16 (66.7)	
German	0 (0)	1 (4.2)	
Italian	2 (8)	3 (12.5)	
English	1 (4)	0 (0)	
Other	10 (40)	4 (16.7)	

a Baseline demographic, clinical, and treatment characteristics of adult outpatients with heart failure with reduced or mildly reduced ejection fraction enrolled at Geneva University Hospitals (Geneva, Switzerland) between March and November 2024, stratified by randomized group (Cardio-Meds intervention vs usual care).

b Baseline characteristics are reported for participants who attended the first study session and completed baseline assessments.

c Baseline group comparisons are presented for descriptive purposes only and should not be interpreted as formal tests of randomization balance.

d HF: heart failure.

e ACE: angiotensin-converting enzyme.

f ARB: angiotensin II receptor blocker.

g ARNI: angiotensin receptor–neprilysin inhibitor.

h BB: beta-blocker.

i MRA: mineralocorticoid receptor antagonist.

j SGLT2: sodium-glucose cotransporter 2.

k AF: atrial fibrillation.

l COPD: chronic obstructive pulmonary disease.

m PAD: peripheral artery disease.

n LVEF: left ventricular ejection fraction.

o NYHA: New York Heart Association.

### App Usage in Test Group

During the 30-day follow-up period, participants in the intervention group used the app for a mean total duration of 177.4 (SD 130.4) minutes, with a mean of 48.6 (SD 36.2) logins. App usage was highly variable across participants. Total usage time had a median of 123 (IQR 74‐273) minutes, and the number of logins had a median of 43 (IQR 19‐85), indicating a skewed distribution with a subset of highly active users. The mean time spent per login was 5.3 (SD 3.6) minutes. Participants spent a mean of 7.9 (SD 9.9) minutes on the information section. Weight was recorded a mean of 5.9 (SD 9.6) times, blood pressure and heart rate were entered a mean of 6.8 (SD 10.3) times, corresponding to an average of approximately 1 entry every 4 to 5 days. Among participants who accessed the quiz feature, a mean of 16.4 (SD 17.6) questions were answered, with a mean of 12.5 (SD 13.9) correct answers, corresponding to a mean accuracy rate of 0.7 (SD 0.3) (Multimedia Appendix 4).

### HF Knowledge and Self-Management (Primary Outcome)

**Figure 9.**
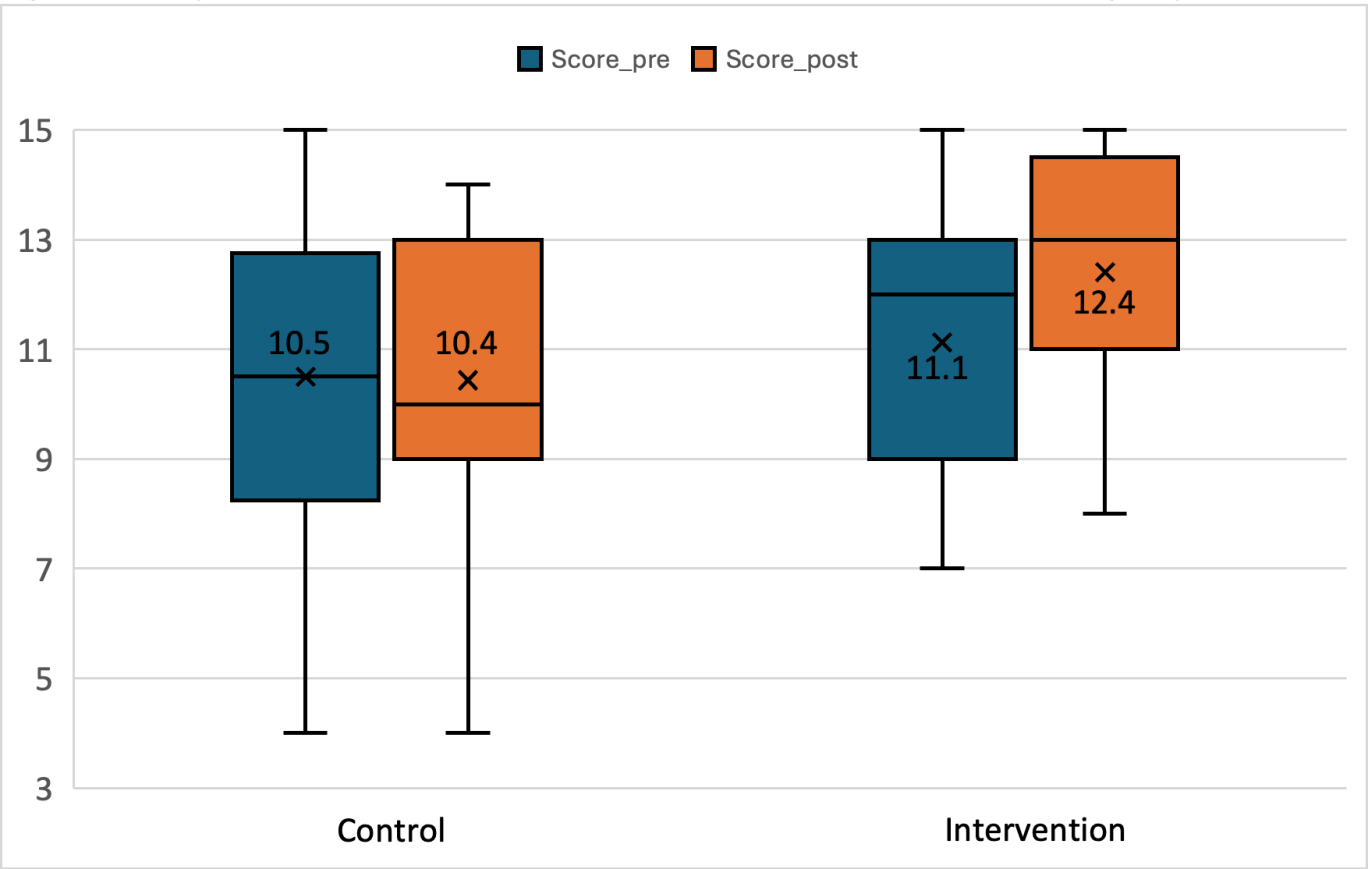
Heart failure knowledge scores before and after a 30-day mobile health intervention*. *Boxplots show Dutch Heart Failure Knowledge Scale (DHFKS) scores at baseline and after 30 days in the intervention group (Cardio-Meds plus usual care) and the control group (usual care alone). The data are derived from a single-center pilot randomized controlled trial conducted at Geneva University Hospitals (Geneva, Switzerland) in adult patients with heart failure between March and November 2024.

The DHFKS questionnaire was administered at both study sessions. At baseline (session 1), the mean score was 11.1 (SD 2.4) in the intervention group and 10.5 (SD 2.9) in the control group. In the primary analysis using ANCOVA adjusted for baseline DHFKS score, the intervention group had significantly higher follow-up knowledge scores than the control group, with an adjusted mean between-group difference of +1.3 points (95% CI 0.40‐2.20; *P*<.001). For descriptive purposes, within-group analyses showed that DHFKS scores remained stable in the control group (10.5 [SD 2.9] at baseline vs 10.4 [SD 3] at follow-up; *P*=.82), whereas the intervention group demonstrated a statistically significant increase (11.1 [SD 2.4] to 12.4 [SD 2.4]; *P*<.001) (Figure 9)(Table 2). Although the improvement in the intervention group was statistically significant (+1.3 points), it did not reach the prespecified +2-point threshold used for sample size estimation. No significant association was observed between app usage metrics (including total usage time and number of logins) and change in DHFKS score .

**Table 2.** Heart failure knowledge before and after a 30-day intervention using the Cardio-Meds mobile app.^a^

Group	Preintervention, mean (SD)	Postintervention, mean (SD)	Mean change	*P* value
Control	10.5 (2.9)	10.4 (3)	–0.1	.82^b^
Intervention	11.1 (2.4)	12.4 (2.4)	1.3	<.001^b^
Adjusted between-group difference (ANCOVA^c^)	—^d^	—^d^	1.3 (95% CI 0.40-2.20)	<.001^e^

a Dutch Heart Failure Knowledge Scale scores at baseline and after 30 days are shown for intervention and control groups in a single-center pilot randomized controlled trial conducted at Geneva University Hospitals (Geneva, Switzerland) among adult patients with heart failure in 2024. Within-group changes and adjusted between-group differences are reported.

b Paired *t* test (within-group).

c ANCOVA: analysis of covariance.

d Not applicable.

e ANCOVA adjusted for baseline DHFKS score.

### Medication Adherence (Secondary Outcome) With BAASIS

For the initiation domain, analyses were restricted to participants with newly prescribed medications during the reference period, resulting in very small sample sizes (control group: n=5 at follow-up; intervention group: n=7). The intervention group maintained a score of 100% at both time points, while the control group showed a decrease from 100% at baseline to 80% at 1 month. The between-group comparison was not statistically significant (*P*=.38); however, this result should be interpreted cautiously given the very limited sample size and lack of statistical power for this specific domain. As per the BAASIS instrument structure, denominators for the “Initiation” domain vary across assessment time points depending on whether participants had newly prescribed medications during the reference period. For the implementation domain, the intervention group showed a decrease from 64% at baseline to 52% at 1 month. In contrast, the control group showed a slight increase, from 46% to 48%, likely due to missing responses from three participants at follow-up. The between-group difference was not statistically significant (*P*=.61). For the persistence domain, the intervention group decreased slightly from 100% to 96% after the intervention, while the control group remained relatively stable, from 96% to 95% (Table 3). These small variations correspond to 1 participant in each group: in the control group, 1 answered “yes” at both time points; in the intervention group, 1 answered “yes” only at the second session. The between-group comparison showed no statistically significant difference (*P*=.76).

**Table 3.** Medication adherence outcomes assessed with the BAASIS questionnaire before and after a 30-day follow-up period.^a^

Domain and group	Preintervention, n/N (%)	Postintervention, n/N (%)	*P* value
Initiation			.38
Control	4/4 (100)	4/5 (80)	
Intervention	14/14 (100)	7/7 (100)	
Implementation			.61
Control	11/24 (46)	10/21 (48)	
Intervention	16/25 (64)	13/25 (52)	
Persistence			.76
Control	23/24 (96)	20/21 (95)	
Intervention	25/25 (100)	24/25 (96)	

a Initiation, implementation, and persistence domains of the Basel Assessment of Adherence to Immunosuppressive Medication Scale (BAASIS) at baseline and 30 days in intervention and control groups. Data derived from a pilot randomized controlled trial evaluating a mobile health intervention in adult outpatients with heart failure conducted at Geneva University Hospitals (Geneva, Switzerland) between March and November 2024. Initiation denominators reflect only participants with newly prescribed medications during the reference period, according to BAASIS specifications.

### Usability of the App (Exploratory Outcome)

**Figure 10.**
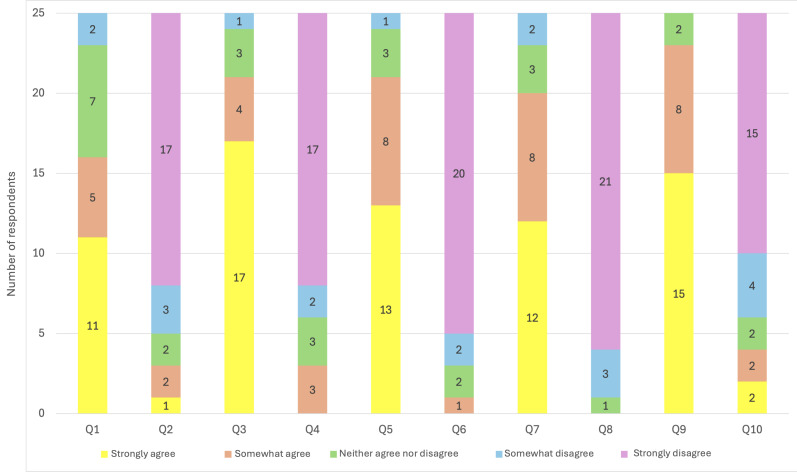
System Usability Scale (SUS) item responses for the Cardio-Meds mobile app. Likert-style plots display responses to the 10 items of the SUS completed after 30 days of app use by participants randomized to the intervention group in a pilot randomized controlled trial. The study was conducted at Geneva University Hospitals (Geneva, Switzerland) between March and November 2024 among adult outpatients with heart failure.

The usability of the app was assessed using the SUS, a standardized questionnaire designed to evaluate user experience and ease of use. The mean SUS score in the intervention group was 84.3 (SD 15) (Multimedia Appendix 5), indicating excellent usability, as it falls within the 90th percentile of usability scores [24]. Notably, 64% (16/25) of participants indicated they would continue using the app (Figure 10). However, beyond the predefined usage metrics collected (eg, total time, logins, section time, and entries), no additional backend analytics were available to characterize navigation pathways or feature-level sequences. Future studies should include backend analytics to better understand interaction dynamics with the app. Across all SUS items, most of the responses reflected positive user experience and good perceived usability of the Cardio-Meds app (Figure 10).

### Participant Feedback

Participants could provide comments at the end of the questionnaires. Overall, the app was seen as well-designed and pleasant to use, with 1 participant describing it as a “companion that sets the rhythm of his day.” Users appreciated features such as the motivational message “Everything is fine today,” medication images for easy identification, and the reminder function. Quizzes and educational content were well received. Reported difficulties included deleting medications after input errors, handling missed doses, and grouping medications, often due to unfamiliarity with the app’s functions. Suggestions included improving text contrast, syncing reminders with smartwatches, managing time zones, integrating with health platforms, storing prescriptions, and adding tracking for weight and blood pressure. Some participants also requested video content and information on disease progression. These comments will inform future app development.

## Discussion

### Principal Findings

In this pilot RCT conducted in a stable ambulatory HF population, a 30-day intervention using the Cardio-Meds mobile app was associated with a statistically significant short-term improvement in HF knowledge and self-management compared with usual care. After adjustment for baseline knowledge, the intervention group demonstrated a statistically significant between-group improvement of 1.3 points in the DHFKS score compared with the control group. The magnitude of improvement, while statistically significant, was smaller than the prespecified effect size and should therefore be interpreted as an exploratory signal rather than a clinically definitive change. In contrast, no significant differences were observed between groups in self-reported medication adherence across the BAASIS domains; small numerical variations should be interpreted cautiously in light of the pilot design and reliance on self-reported measures. The app demonstrated high usability and acceptability, with excellent SUS scores and a majority of participants reporting willingness to continue using the app. Taken together, these findings should be interpreted as short-term, exploratory results primarily informing feasibility, usability, and educational impact rather than durable behavioral change.

### Comparison With Prior Work

Previous digital health interventions for HF have reported mixed but generally encouraging effects on self-care behaviors and symptom outcomes. For example, Yoon et al [14] found that a smartphone-based intervention with remote feedback improved HF symptom burden over follow-up, and Kitsiou et al [13] showed that multiapp interventions may enhance self-care engagement. Systematic evidence also supports the value of digital health tools in HF management, reinforcing the need for continued innovation and larger, long-term studies [15]. In this context, the improvement in HF knowledge observed in our study is consistent with prior evidence showing that digital interventions can enhance patient understanding and engagement. A recent systematic review by Mouselimis et al [25] reported that mHealth tools frequently improve disease knowledge and engagement, although effects on clinical outcomes and adherence remain heterogeneous. Fernández-Gutiérrez et al [20] emphasized the importance of validated, patient-centered educational content in HF apps, an aspect directly addressed in Cardio-Meds through guideline-based information and interactive quizzes. Similarly, Choi et al [26] demonstrated that an HF-specific mobile app improved functional and echocardiographic parameters, although such clinical endpoints were not assessed in the present study. Unlike the findings of Ruppar et al [10], who reported improvements in medication adherence following adherence-focused interventions, we did not observe a significant adherence effect. This discrepancy may partly be explained by the characteristics of the study population. Baseline adherence was high in the persistence domain, with nearly all participants reporting continued use of prescribed medications, reflecting a stable ambulatory population with established treatment routines. In contrast, baseline adherence in the implementation domain was suboptimal in both groups, indicating difficulties related to day-to-day medication-taking behavior rather than treatment discontinuation. Despite this, no significant improvement in implementation adherence was observed after the 30-day intervention, possibly due to the short follow-up period, reliance on self-reported measures, and the exploratory nature of the study. Usability and acceptability are critical determinants of digital intervention success, and Cardio-Meds performed well in this regard, with a mean SUS score of 84, consistent with excellent usability [24] and comparable to other successful HF apps.

### Limitations

Several limitations should be acknowledged. First, this pilot study was powered exclusively for the primary outcome of HF knowledge. Consequently, it was underpowered to detect differences in secondary outcomes such as medication adherence, particularly in a stable ambulatory population with high baseline adherence. Adherence findings should therefore be interpreted as exploratory and hypothesis-generating rather than confirmatory.

Second, the short follow-up period limited assessment of longer-term knowledge retention, sustained behavioral change, and clinical outcomes. Short-term improvements may partly reflect transient or “honeymoon” effects, and longer follow-up will be required to evaluate the durability of effects on self-management and adherence.

Third, medication adherence was assessed using self-reported measures, which are inherently subject to recall and social desirability bias. Although validated instruments were used, future studies would benefit from incorporating objective adherence measures such as pharmacy refill data or electronic monitoring. In this context, the small, non-significant decrease in implementation adherence observed in the intervention group may reflect increased awareness of medication-taking behavior and more accurate self-reporting following exposure to the intervention and repeated questioning, rather than a true deterioration in adherence.

Fourth, the single-center design and inclusion of a stable, chronically treated outpatient population may limit generalizability, particularly to recently hospitalized or higher-risk patients.

Fifth, HF knowledge was assessed using a French translation of the DHFKS that did not undergo formal linguistic or psychometric validation. While the items are factual and clinically straightforward and the original scale was developed in a population comparable to the Swiss context, this limits the interpretation of absolute score values and the strength of conclusions regarding knowledge gains. In addition, the absence of a validated minimal clinically important difference for the DHFKS constrains assessment of the clinical relevance of the observed improvement. Accordingly, knowledge findings should be considered exploratory.

Sixth, app engagement showed marked heterogeneity, with a small number of highly active users and overall limited use of specific features such as physiological self-monitoring. This skewed distribution limits the interpretation of average usage statistics and precluded meaningful dose-response analyses. Consistent with this, no clear association was observed between engagement metrics and improvement in knowledge scores. Although a testing effect from repeated questionnaire administration may have contributed to knowledge gains, this would be expected to affect both groups similarly, and the absence of improvement in the control group suggests that testing alone is unlikely to explain the observed between-group difference.

Finally, the intervention group received a brief mid-study technical support call that was not mirrored in the control group. Although this contact was limited to troubleshooting and did not include educational or behavioral guidance, an attention-related or Hawthorne effect cannot be fully excluded. In addition, baseline imbalances were observed between groups, with the intervention group being younger and including more non-native French speakers. Such differences may occur by chance in small pilot studies and could have influenced engagement, as age may proxy digital literacy. Given the limited sample size, post hoc adjustment was not performed, and these factors should be considered potential confounders.

### Future Directions

Future research should evaluate Cardio-Meds in larger, multicenter randomized trials with longer follow-up and inclusion of clinical endpoints such as hospitalization, emergency visits, or quality of life. Studying the intervention in the early post-discharge period, when patients are particularly vulnerable and educational needs are high, may be especially informative. Further development could also include adaptation to different literacy levels and languages to enhance accessibility and equity of care.

### Conclusions

In this pilot RCT, use of the Cardio-Meds mobile app was associated with a statistically significant but modest short-term improvement in HF knowledge compared with usual care, without measurable changes in self-reported medication adherence. The app demonstrated high usability and acceptability in a stable ambulatory HF population. These findings should be interpreted as exploratory and hypothesis-generating and support further evaluation of Cardio-Meds in larger studies with longer follow-up and clinically relevant outcomes.

## Supplementary material

10.2196/83022 Multimedia Appendix 1 Dutch Heart Failure Knowledge Scale questionnaire translated in French with DeepL.

10.2196/83022 Multimedia Appendix 2 Basel Assessment of Adherence to Immunosuppressive Medications Scale (BAASIS) in French.

10.2196/83022 Multimedia Appendix 3 Usability questionnaire.

10.2196/83022 Multimedia Appendix 4 Usage of the app in the intervention group during the 30 days follow-up.

10.2196/83022 Multimedia Appendix 5 System Usability Scale (SUS) questionnaire responses.

10.2196/83022 Checklist 1 CONSORT checklist.

## Abbreviations

BAASIS: Basel Assessment of Adherence to Immunosuppressive Medication Scale
DHFKS: Dutch Heart Failure Knowledge Scale
HF: heart failure
HFmrEF: heart failure with mildly reduced ejection fraction
HFrEF: heart failure with reduced ejection fraction
HUG: Geneva University Hospitals
mHealth: mobile health
RCT: randomized controlled trial
SUS: System Usability Scale
