# Supplementary material for: Impact of the Cardio-Meds Mobile App on Heart Failure Knowledge and Medication Adherence: Pilot Randomized Controlled Trial
Source: JMIR Cardio. 2026 Feb 23;10:e83022. doi: 10.2196/83022 (PMC12928692; doi:10.2196/83022)
Supplement: Multimedia Appendix 1 [file cardio-v10-e83022-s001.docx]

## **Appendix 1** Dutch Heart Failure Knowledge Scale questionnaire translated in French with DeepL

1. À quelle fréquence les patients souffrant d'insuffisance cardiaque sévère doivent-ils se peser ?

❑ chaque semaine

❑ de temps en temps

❑ tous les jours

1. Pourquoi est-il important que les patients souffrant d'insuffisance cardiaque se pèsent régulièrement ?

❑ parce que de nombreux patients souffrant d'insuffisance cardiaque ont peu d'appétit

❑ pour vérifier si le corps retient les liquides

❑ pour évaluer la bonne dose de médicaments

1. Quelle quantité de liquide pouvez-vous prendre à la maison chaque jour ?

❑ 1,5 à 2,5 litres au maximum

❑ Le moins de liquide possible

❑ autant de liquide que possible

4. Laquelle de ces affirmations est vraie ?

❑ Lorsque je tousse beaucoup, il vaut mieux que je ne prenne pas mon médicament contre l'insuffisance cardiaque.

❑ Lorsque je me sens mieux, je peux arrêter de prendre mon médicament contre l'insuffisance cardiaque.

❑ Il est important que je prenne régulièrement mon médicament contre l'insuffisance cardiaque.

5. Quelle est la meilleure chose à faire en cas d'essoufflement accru ou de jambes gonflées ?

❑ appeler le médecin ou l'infirmière

❑ attendre le prochain contrôle

❑ prendre moins de médicaments

6. Qu'est-ce qui peut provoquer une aggravation rapide des symptômes de l'insuffisance cardiaque ?

❑ un régime alimentaire riche en graisses

❑ un rhume ou une grippe

❑ le manque d'exercice

7. Que signifie l'insuffisance cardiaque ?

❑ que le cœur est incapable de pomper suffisamment de sang dans l'organisme

❑ qu'une personne ne fait pas assez d'exercice et qu'elle est en mauvaise condition physique

❑ qu'il y a un caillot de sang dans les vaisseaux sanguins du cœur.

8. Pourquoi les jambes peuvent-elles gonfler en cas d'insuffisance cardiaque ?

❑ parce que les valves des vaisseaux sanguins des jambes ne fonctionnent pas correctement

❑ parce que les muscles des jambes ne reçoivent pas assez d'oxygène

❑ à cause de l'accumulation de liquide dans les jambes

9. Quelle est la fonction du cœur ?

❑ absorber les nutriments du sang

❑ pomper le sang dans tout le corps

❑ Fournir de l'oxygène au sang

10. Pourquoi une personne souffrant d'insuffisance cardiaque doit-elle suivre un régime pauvre en sel ?

❑ Le sel favorise la rétention d'eau.

❑ Le sel provoque une constriction des vaisseaux sanguins.

❑ Le sel augmente la fréquence cardiaque

11. Quelles sont les principales causes de l'insuffisance cardiaque ?

❑ un infarctus du myocarde et l'hypertension artérielle

❑ les problèmes pulmonaires et l'allergie

❑ l'obésité et le diabète

12. Quelle affirmation concernant l'exercice pour les personnes souffrant d'insuffisance cardiaque est vraie ?

❑ Il est important de faire le moins d'exercice possible à la maison pour soulager le cœur.

❑ Il est important de faire de l'exercice à la maison et de se reposer régulièrement entre les deux.

❑ il est important de faire le plus d'exercice possible à la maison

13. Pourquoi des comprimés diurétiques sont-ils prescrits à une personne souffrant d'insuffisance cardiaque ?

❑ pour abaisser la tension artérielle

❑ pour prévenir la rétention d'eau dans l'organisme

❑ parce qu'ils peuvent ainsi boire davantage

14. Quelle affirmation concernant l'augmentation du poids et l'insuffisance cardiaque est vraie ?

❑ Une augmentation de plus de 2 kg en 2 ou 3 jours doit être signalée au médecin lors du prochain contrôle.

❑ en cas d'augmentation de plus de 2 kg en 2 ou 3 jours, il faut contacter le médecin ou l'infirmière

❑ en cas d'augmentation de plus de 2 kg en 2 ou 3 jours, vous devez manger moins.

15. Quelle est la meilleure chose à faire quand on a soif ?

❑ sucer un glaçon

❑ sucer une pastille

❑ boire beaucoup
