# Supplementary material for: Impact of the Cardio-Meds Mobile App on Heart Failure Knowledge and Medication Adherence: Pilot Randomized Controlled Trial
Source: JMIR Cardio. 2026 Feb 23;10:e83022. doi: 10.2196/83022 (PMC12928692; doi:10.2196/83022)
Supplement: Multimedia Appendix 2 [file cardio-v10-e83022-s002.docx]

## **Appendix 2** Basel Assessment of Adherence to ImmunoSuppressive Medications Scale (BAASIS®) in French

| 1A. Avez-vous omis de prendre vos médicaments pour l’insuffisance cardiaque, ne serait-ce qu’une seule fois au cours des 4 dernières semaines ? | Oui  Non | **Si oui**, combien de fois cela s’est-il produit au cours des 4 dernières semaines ?   - Une fois - Deux fois - Trois fois - Quatre fois - Plus de 4 fois |
| --- | --- | --- |
| 1B. **Si vous avez répondu oui à la question 1A**, avez-vous déjà sauté deux doses ou plus d’affilée au cours des 4 dernières semaines ? | Oui  Non | **Si oui**, combien de fois cela s’est-il produit au cours des 4 dernières semaines ?   - Une fois - Deux fois - Trois fois - Quatre fois - Plus de 4 fois |
| 2. Avez-vous pris l’un de vos médicaments pour l’insuffisance cardiaque plus de 2 heures avant ou après l’heure à laquelle vous les prenez habituellement au cours des 4 dernières semaines ? | Oui  Non | **Si oui**, combien de fois cela s’est-il produit au cours des 4 dernières semaines ?   - Une fois - Deux fois - Trois fois - Quatre fois - Plus de 4 fois |
| 3. Avez-vous modifié la quantité prescrite de l'un ou l'autre de vos médicaments pour l’insuffisance cardiaque au cours des 4 dernières semaines, de votre propre initiative, sans que votre médecin ne vous l'ait demandé ? Par exemple, avez-vous pris plus ou moins de pilules ou changé la dose, peut-être en coupant une pilule en deux ? | Oui  Non |  |
| 4. Avez-vous complètement cessé de prendre l’un ou l'autre de vos médicaments pour l’insuffisance cardiaque au cours de la dernière année, de votre propre initiative, sans que votre médecin ne vous l'ait demandé ? | Oui  Non |  |
| 5. Votre médecin de famille ou votre spécialiste vous a-t-il prescrit de nouveaux médicaments au cours de la dernière année ? (Peut-être des médicaments contre l'hypertension artérielle ou des médicaments pour réduire le cholestérol ou les lipides ?) | Oui  Non | **Si oui**, avez-vous donné l’ordonnance à la pharmacie et commencé à prendre ce nouveau médicament ?   - Oui - Non |
