# Supplementary material for: Impact of the Cardio-Meds Mobile App on Heart Failure Knowledge and Medication Adherence: Pilot Randomized Controlled Trial
Source: JMIR Cardio. 2026 Feb 23;10:e83022. doi: 10.2196/83022 (PMC12928692; doi:10.2196/83022)
Supplement: Multimedia Appendix 3 [file cardio-v10-e83022-s003.docx]

## **Appendix 3** Usability questionnaire
