# Supplementary material for: Impact of the Cardio-Meds Mobile App on Heart Failure Knowledge and Medication Adherence: Pilot Randomized Controlled Trial
Source: JMIR Cardio. 2026 Feb 23;10:e83022. doi: 10.2196/83022 (PMC12928692; doi:10.2196/83022)
Supplement: Multimedia Appendix 4 [file cardio-v10-e83022-s004.docx]

Usage of the application in the intervention group during the 30 days follow-up

|  | **Total duration (min)** | **Number of logins** | **Average time per login (min)** | **Time on info section (min)** | **Vital signs entries** | | **Total quiz answers** | **Correct answers** | **Percentage of correct answers** |
| --- | --- | --- | --- | --- | --- | --- | --- | --- | --- |
|  |  |  |  |  | **Weight** | **BP & HR** |  |  |  |
| P1 | 229 | 92 | 2,5 | 12 | 14 | 12 |  |  |  |
| P2 | 123 | 16 | 7,7 | 2 | 2 | 0 | 1 | 1 | 100,0% |
| P3 | 94 | 62 | 1,5 | 18 | 0 | 0 | 4 | 4 | 100,0% |
| P4 | 498 | 85 | 5,9 | 1 | 7 | 5 | 47 | 33 | 70,2% |
| P5 | 60 | 6 | 10,0 | 1 | 3 | 2 |  |  |  |
| P6 | 90 | 10 | 9,0 | 6 | 4 | 0 |  |  |  |
| P7 | 331 | 112 | 3,0 | 6 | 34 | 25 | 37 | 27 | 73,0% |
| P8 | 265 | 87 | 3,0 | 1 | 5 | 0 |  |  |  |
| P9 | 74 | 7 | 10,6 | 25 | 2 | 1 | 3 | 2 | 66,7% |
| P10 | 46 | 7 | 6,6 | 1 | 0 | 0 |  |  |  |
| P11 | 291 | 105 | 2,8 | 6 | 0 | 1 | 16 | 13 | 81,3% |
| P12 | 457 | 91 | 5,0 | 11 | 0 | 0 | 1 | 0 | 0,0% |
| P13 | 87 | 97 | 0,9 | 8 | 0 | 0 |  |  |  |
| P14 | 46 | 3 | 15,3 | 0 | 3 | 2 |  |  |  |
| P15 | 126 | 19 | 6,6 | 42 | 2 | 5 | 6 | 3 | 50,0% |
| P16 | 71 | 53 | 1,3 | 11 | 0 | 24 | 9 | 9 | 100,0% |
| P17 | 96 | 25 | 3,8 | 4 | 1 | 1 | 3 | 1 | 33,3% |
| P18 | 273 | 54 | 5,1 | 3 | 1 | 0 |  |  |  |
| P19 | 45 | 24 | 1,9 | 0 | 9 | 8 |  |  |  |
| P20 | 217 | 20 | 10,9 | 2 | 3 | 2 | 13 | 7 | 53,8% |
| P21 | 291 | 70 | 4,2 | 1 | 26 | 26 | 54 | 47 | 87,0% |
| P22 | 177 | 43 | 4,1 | 2 | 0 | 0 | 26 | 19 | 73,1% |
| P23 | 110 | 19 | 5,8 | 13 | 1 | 0 | 34 | 25 | 73,5% |
| P24 | 48 | 33 | 1,5 | 0 | 1 | 28 | 3 | 3 | 100,0% |
| P25 | 289 | 76 | 3,8 | 21 | 29 | 28 | 6 | 6 | 100,0% |
| **Mean** | **177,4** | **48,6** | **5,3** | **7,9** | **5,9** | **6,8** | **16,4** | **12,5** | **0,7** |
| **(SD)** | **(130,4)** | **(36,2)** | **(3,6)** | **(9,9)** | **(9,6)** | **(10,3)** | **(17,6)** | **(13,9)** | **(0,3)** |

P = Participant; min = minutes; BP = Blood Pressure; HR = Heart Rate; SD = Standard Deviation
