# Supplementary material for: Impact of the Cardio-Meds Mobile App on Heart Failure Knowledge and Medication Adherence: Pilot Randomized Controlled Trial
Source: JMIR Cardio. 2026 Feb 23;10:e83022. doi: 10.2196/83022 (PMC12928692; doi:10.2196/83022)
Supplement: Multimedia Appendix 5 [file cardio-v10-e83022-s005.docx]

System Usability Scale (SUS) questionnaire responses

|  | **Q1** | **Q2** | **Q3** | **Q4** | **Q5** | **Q6** | **Q7** | **Q8** | **Q9** | **Q10** | **SUS Score**  **(Sumx2,5)** |
| --- | --- | --- | --- | --- | --- | --- | --- | --- | --- | --- | --- |
| **Participant 1** | 4 | 4 | 4 | 4 | 4 | 4 | 4 | 4 | 4 | 4 | 100 |
| **Participant 2** | 4 | 4 | 4 | 4 | 4 | 4 | 3 | 4 | 4 | 3 | 95 |
| **Participant 3** | 4 | 4 | 4 | 4 | 4 | 4 | 4 | 4 | 4 | 4 | 100 |
| **Participant 4** | 3 | 3 | 3 | 2 | 3 | 4 | 3 | 3 | 3 | 2 | 72,5 |
| **Participant 5** | 2 | 4 | 4 | 4 | 4 | 4 | 4 | 4 | 4 | 4 | 95 |
| **Participant 6** | 1 | 4 | 4 | 4 | 4 | 4 | 4 | 4 | 4 | 4 | 92,5 |
| **Participant 7** | 2 | 4 | 4 | 4 | 4 | 4 | 4 | 3 | 3 | 4 | 90 |
| **Participant 8** | 4 | 4 | 4 | 4 | 4 | 4 | 4 | 4 | 4 | 4 | 100 |
| **Participant 9** | 4 | 4 | 4 | 4 | 4 | 4 | 4 | 4 | 4 | 4 | 100 |
| **Participant 10** | 4 | 0 | 4 | 1 | 4 | 4 | 3 | 4 | 4 | 3 | 77,5 |
| **Participant 11** | 3 | 3 | 3 | 4 | 3 | 4 | 3 | 4 | 3 | 4 | 85 |
| **Participant 12** | 4 | 1 | 3 | 1 | 4 | 3 | 4 | 4 | 4 | 0 | 70 |
| **Participant 13** | 3 | 1 | 4 | 4 | 3 | 4 | 4 | 4 | 4 | 4 | 87,5 |
| **Participant 14** | 2 | 2 | 2 | 4 | 2 | 3 | 4 | 4 | 3 | 1 | 67,5 |
| **Participant 15** | 2 | 4 | 4 | 4 | 3 | 4 | 4 | 4 | 4 | 4 | 92,5 |
| **Participant 16** | 3 | 4 | 4 | 4 | 3 | 4 | 1 | 4 | 4 | 4 | 87,5 |
| **Participant 17** | 2 | 2 | 2 | 2 | 2 | 2 | 2 | 2 | 2 | 2 | 50 |
| **Participant 18** | 3 | 4 | 1 | 1 | 1 | 1 | 1 | 4 | 3 | 4 | 57,5 |
| **Participant 19** | 2 | 3 | 2 | 3 | 3 | 2 | 3 | 3 | 2 | 1 | 60 |
| **Participant 20** | 2 | 4 | 3 | 3 | 3 | 4 | 3 | 4 | 3 | 3 | 80 |
| **Participant 21** | 4 | 4 | 4 | 4 | 4 | 4 | 2 | 4 | 3 | 4 | 92,5 |
| **Participant 22** | 1 | 4 | 4 | 2 | 2 | 4 | 2 | 4 | 4 | 0 | 67,5 |
| **Participant 23** | 4 | 4 | 4 | 4 | 4 | 4 | 4 | 4 | 4 | 4 | 100 |
| **Participant 24** | 4 | 4 | 4 | 4 | 3 | 4 | 3 | 4 | 3 | 3 | 90 |
| **Participant 25** | 4 | 4 | 4 | 4 | 4 | 4 | 3 | 4 | 4 | 4 | 97,5 |
| **Mean**  **(SD)** | **3,0**  **(1,0)** | **3,3**  **(1,2)** | **3,5**  **(0,9)** | **3,3**  **(1,1)** | **3,3**  **(0,9)** | **3,6**  **(0,8)** | **3,2**  **(1,0)** | **3,8**  **(0,5)** | **3,5**  **(0,7)** | **3,1**  **(1,3)** | **84,3**  **(15,0)** |

Q = Question; SUS = System Usability Scale; SD = Standard Deviation; Mean = Moyenne
