# Supplementary material for: Impact of the Cardio-Meds Mobile App on Heart Failure Knowledge and Medication Adherence: Pilot Randomized Controlled Trial
Source: JMIR Cardio. 2026 Feb 23;10:e83022. doi: 10.2196/83022 (PMC12928692; doi:10.2196/83022)
Supplement: Checklist 1 [file cardio-v10-e83022-s006.pdf]

CONSORT-EHEALTH (V 1.6.1) - Submission/Publication Form

The CONSORT-EHEALTH checklist is intended for authors of randomized trials evaluating web-based and Internet-based applications/interventions, including mobile interventions, electronic games (incl multiplayer games), social media, certain telehealth applications, and other interactive and/or networked electronic applications. Some of the items (e.g. all subitems under item 5 - description of the intervention) may also be applicable for other study designs.

The goal of the CONSORT EHEALTH checklist and guideline is to be
a) a guide for reporting for authors of RCTs,
b) to form a basis for appraisal of an ehealth trial (in terms of validity)

CONSORT-EHEALTH items/subitems are MANDATORY reporting items for studies published in the Journal of Medical Internet Research and other journals / scientific societies endorsing the checklist.

Items numbered 1., 2., 3., 4a., 4b etc are original CONSORT or CONSORT-NPT (non-pharmacologic treatment) items.
Items with Roman numerals (i., ii, iii, iv etc.) are CONSORT-EHEALTH extensions/clarifications.

As the CONSORT-EHEALTH checklist is still considered in a formative stage, we would ask that you also RATE ON A SCALE OF 1-5 how important/useful you feel each item is FOR THE PURPOSE OF THE CHECKLIST and reporting guideline (optional).

Mandatory reporting items are marked with a red *.
In the textboxes, either copy & paste the relevant sections from your manuscript into this form - please include any quotes from your manuscript in QUOTATION MARKS,
or answer directly by providing additional information not in the manuscript, or elaborating on why the item was not relevant for this study.

YOUR ANSWERS WILL BE PUBLISHED AS A SUPPLEMENTARY FILE TO YOUR PUBLICATION IN JMIR AND ARE CONSIDERED PART OF YOUR PUBLICATION (IF ACCEPTED).
Please fill in these questions diligently. Information will not be copyedited, so please use proper spelling and grammar, use correct capitalization, and avoid abbreviations.

DO NOT FORGET TO SAVE AS PDF _AND_ CLICK THE SUBMIT BUTTON SO YOUR ANSWERS ARE IN OUR DATABASE !!!

Citation Suggestion (if you append the pdf as Appendix we suggest to cite this paper in the caption):
Eysenbach G, CONSORT-EHEALTH Group
CONSORT-EHEALTH: Improving and Standardizing Evaluation Reports of Web-based and Mobile Health Interventions
J Med Internet Res 2011;13(4):e126
URL: http://www.jmir.org/2011/4/e126/
doi: 10.2196/jmir.1923
PMID: 22209829

victor.buswell@gmail.com Changer de compte

Non partagé

Enregistrement…

* Indique une question obligatoire

Your name *

First Last

Victor Buswell

Primary Affiliation (short), City, Country *

University of Toronto, Toronto, Canada

Geneva University Hospitals, Geneva, Switzerla

Your e-mail address *

abc@gmail.com

victor.buswell@gmail.com

Title of your manuscript *

Provide the (draft) title of your manuscript.

Impact of the Cardio-Meds mobile app on heart failure knowledge and medication adherence: a pilot randomized controlled trial

Name of your App/Software/Intervention *

If there is a short and a long/alternate name, write the short name first and add the long name in brackets.

Swiss-Meds

Evaluated Version (if any)

e.g. "V1", "Release 2017-03-01", "Version 2.0.27913"

CardioMeds

Language(s) *

What language is the intervention/app in? If multiple languages are available, separate by comma (e.g. "English, French")

French, German

URL of your Intervention Website or App

e.g. a direct link to the mobile app on app in appstore (itunes, Google Play), or URL of the website. If the intervention is a DVD or hardware, you can also link to an Amazon page.

https://www.google.com/url?sa=t&source=web&rct=j&opi=89978449&url=https://apps.apple

URL of an image/screenshot (optional)

Votre réponse

Accessibility *

Can an enduser access the intervention presently?

- (•) access is free and open
- ( ) access only for special usergroups, not open
- ( ) access is open to everyone, but requires payment/subscription/in-app purchases
- ( ) app/intervention no longer accessible
- ( ) Autre :

Primary Medical Indication/Disease/Condition *

e.g. "Stress", "Diabetes", or define the target group in brackets after the condition, e.g. "Autism (Parents of children with)", "Alzheimers (Informal Caregivers of)"

Heart Failure

Primary Outcomes measured in trial *

comma-separated list of primary outcomes reported in the trial

Impact of a 30-day Cardio-Meds intervention o

Secondary/other outcomes

Are there any other outcomes the intervention is expected to affect?

Impact of a 30-day Cardio-Meds intervention on medication adherence
Usability of Cardio-Meds application

Recommended "Dose" *

What do the instructions for users say on how often the app should be used?

- (x) Approximately Daily
- ( ) Approximately Weekly
- ( ) Approximately Monthly
- ( ) Approximately Yearly
- ( ) "as needed"
- ( ) Autre :

Approx. Percentage of Users (starters) still using the app as recommended after 3 months *

- (•) unknown / not evaluated
- ( ) 0-10%
- ( ) 11-20%
- ( ) 21-30%
- ( ) 31-40%
- ( ) 41-50%
- ( ) 51-60%
- ( ) 61-70%
- ( ) 71%-80%
- ( ) 81-90%
- ( ) 91-100%
- ( ) Autre :

Overall, was the app/intervention effective? *

- (x) yes: all primary outcomes were significantly better in intervention group vs control
- ( ) partly: SOME primary outcomes were significantly better in intervention group vs control
- ( ) no statistically significant difference between control and intervention
- ( ) potentially harmful: control was significantly better than intervention in one or more outcomes
- ( ) inconclusive: more research is needed
- ( ) Autre :

Article Preparation Status/Stage *

At which stage in your article preparation are you currently (at the time you fill in this form)

- ( ) not submitted yet - in early draft status
- ( ) not submitted yet - in late draft status, just before submission
- ( ) submitted to a journal but not reviewed yet
- (x) submitted to a journal and after receiving initial reviewer comments
- ( ) submitted to a journal and accepted, but not published yet
- ( ) published
- ( ) Autre :

Journal *

If you already know where you will submit this paper (or if it is already submitted), please provide the journal name (if it is not JMIR, provide the journal name under "other")

- ○ not submitted yet / unclear where I will submit this
- ○ Journal of Medical Internet Research (JMIR)
- ○ JMIR mHealth and UHealth
- ○ JMIR Serious Games
- ○ JMIR Mental Health
- ○ JMIR Public Health
- ○ JMIR Formative Research
- ◉ Other JMIR sister journal
- ○ Autre :

Is this a full powered effectiveness trial or a pilot/feasibility trial? *

- ◉ Pilot/feasibility
- ○ Fully powered

Manuscript tracking number *

If this is a JMIR submission, please provide the manuscript tracking number under "other" (The ms tracking number can be found in the submission acknowledgement email, or when you login as author in JMIR. If the paper is already published in JMIR, then the ms tracking number is the four-digit number at the end of the DOI, to be found at the bottom of each published article in JMIR)

- ○ no ms number (yet) / not (yet) submitted to / published in JMIR
- ◉ Autre : 83022

TITLE AND ABSTRACT

1a) TITLE: Identification as a randomized trial in the title

1a) Does your paper address CONSORT item 1a? *

I.e does the title contain the phrase "Randomized Controlled Trial"? (if not, explain the reason under "other")

- ◉ yes
- ○ Autre :

1a-i) Identify the mode of delivery in the title

Identify the mode of delivery. Preferably use “web-based” and/or “mobile” and/or “electronic game” in the title. Avoid ambiguous terms like “online”, “virtual”, “interactive”. Use “Internet-based” only if Intervention includes non-web-based Internet components (e.g. email), use “computer-based” or “electronic” only if offline products are used. Use “virtual” only in the context of “virtual reality” (3-D worlds). Use “online” only in the context of “online support groups”. Complement or substitute product names with broader terms for the class of products (such as “mobile” or “smart phone” instead of “iphone”), especially if the application runs on different platforms.

	1	2	3	4	5	
subitem not at all important	○	○	○	○	○	essential

Does your paper address subitem 1a-i? *

Copy and paste relevant sections from manuscript title (include quotes in quotation marks "like this" to indicate direct quotes from your manuscript), or elaborate on this item by providing additional information not in the ms, or briefly explain why the item is not applicable/relevant for your study

"Impact of the Cardio-Meds mobile app on heart failure knowledge and medication adherence: a pilot randomized controlled trial"

1a-ii) Non-web-based components or important co-interventions in title

Mention non-web-based components or important co-interventions in title, if any (e.g., “with telephone support”).

	1	2	3	4	5	
subitem not at all important	○	○	○	○	○	essential

Does your paper address subitem 1a-ii?

Copy and paste relevant sections from manuscript title (include quotes in quotation marks "like this" to indicate direct quotes from your manuscript), or elaborate on this item by providing additional information not in the ms, or briefly explain why the item is not applicable/relevant for your study

Votre réponse

1a-iii) Primary condition or target group in the title

Mention primary condition or target group in the title, if any (e.g., “for children with Type I Diabetes”) Example: A Web-based and Mobile Intervention with Telephone Support for Children with Type I Diabetes: Randomized Controlled Trial

	1	2	3	4	5	
subitem not at all important	○	○	○	○	○	essential

Does your paper address subitem 1a-iii? *

Copy and paste relevant sections from manuscript title (include quotes in quotation marks "like this" to indicate direct quotes from your manuscript), or elaborate on this item by providing additional information not in the ms, or briefly explain why the item is not applicable/relevant for your study

"Impact of the Cardio-Meds mobile app on heart failure knowledge and medication adherence: a pilot randomized controlled trial"

1b) ABSTRACT: Structured summary of trial design, methods, results, and conclusions

NPT extension: Description of experimental treatment, comparator, care providers, centers, and blinding status.

1b-i) Key features/functionalities/components of the intervention and comparator in the METHODS section of the ABSTRACT

Mention key features/functionalities/components of the intervention and comparator in the abstract. If possible, also mention theories and principles used for designing the site. Keep in mind the needs of systematic reviewers and indexers by including important synonyms. (Note: Only report in the abstract what the main paper is reporting. If this information is missing from the main body of text, consider adding it)

	1	2	3	4	5	
subitem not at all important	○	○	○	○	○	essential

Does your paper address subitem 1b-i? *

Copy and paste relevant sections from the manuscript abstract (include quotes in quotation marks "like this" to indicate direct quotes from your manuscript), or elaborate on this item by providing additional information not in the ms, or briefly explain why the item is not applicable/relevant for your study

"Cardio-Meds is a mobile application developed at Geneva University Hospitals to support HF self-management through structured educational content, interactive quizzes with feedback, medication lists with optional reminders and intake confirmation, and tools for monitoring weight and vital signs."
"Participants were randomized to Cardio-Meds use for 30 days plus usual care or to usual care alone."

1b-ii) Level of human involvement in the METHODS section of the ABSTRACT

Clarify the level of human involvement in the abstract, e.g., use phrases like “fully automated” vs. “therapist/nurse/care provider/physician-assisted” (mention number and expertise of providers involved, if any). (Note: Only report in the abstract what the main paper is reporting. If this information is missing from the main body of text, consider adding it)

	1	2	3	4	5	
subitem not at all important	○	○	○	○	○	essential

Does your paper address subitem 1b-ii?

Copy and paste relevant sections from the manuscript abstract (include quotes in quotation marks "like this" to indicate direct quotes from your manuscript), or elaborate on this item by providing additional information not in the ms, or briefly explain why the item is not applicable/relevant for your study

"Participants were randomized to Cardio-Meds use for 30 days, a self-guided intervention with a single standardized technical support call, plus usual care or to usual care alone."

1b-iii) Open vs. closed, web-based (self-assessment) vs. face-to-face assessments in the METHODS section of the ABSTRACT

Mention how participants were recruited (online vs. offline), e.g., from an open access website or from a clinic or a closed online user group (closed usergroup trial), and clarify if this was a purely web-based trial, or there were face-to-face components (as part of the intervention or for assessment). Clearly say if outcomes were self-assessed through questionnaires (as common in web-based trials). Note: In traditional offline trials, an open trial (open-label trial) is a type of clinical trial in which both the researchers and participants know which treatment is being administered. To avoid confusion, use “blinded” or “unblinded” to indicated the level of blinding instead of “open”, as “open” in web-based trials usually refers to “open access” (i.e. participants can self-enrol). (Note: Only report in the abstract what the main paper is reporting. If this information is missing from the main body of text, consider adding it)

	1	2	3	4	5	
subitem not at all important	○	○	○	○	○	essential

Does your paper address subitem 1b-iii?

Copy and paste relevant sections from the manuscript abstract (include quotes in quotation marks "like this" to indicate direct quotes from your manuscript), or elaborate on this item by providing additional information not in the ms, or briefly explain why the item is not applicable/relevant for your study

"Participants recruited by phone and were randomized to Cardio-Meds use for 30 days, a self-guided intervention with a single standardized technical support call, plus usual care or to usual care alone. The outcomes were self-assessed using standardized questionnaires"

1b-iv) RESULTS section in abstract must contain use data

Report number of participants enrolled/assessed in each group, the use/uptake of the intervention (e.g., attrition/adherence metrics, use over time, number of logins etc.), in addition to primary/secondary outcomes. (Note: Only report in the abstract what the main paper is reporting. If this information is missing from the main body of text, consider adding it)

	1	2	3	4	5	
subitem not at all important	○	○	○	○	○	essential

Does your paper address subitem 1b-iv?

Copy and paste relevant sections from the manuscript abstract (include quotes in quotation marks "like this" to indicate direct quotes from your manuscript), or elaborate on this item by providing additional information not in the ms, or briefly explain why the item is not applicable/relevant for your study

"A total of 49 participants were included (25 intervention, 24 control; 78% male; mean age 62±11.4 years). In intervention group, median app usage was 123 minutes (IQR 74–273), with median of 43 logins (IQR 19–85). "

1b-v) CONCLUSIONS/DISCUSSION in abstract for negative trials

Conclusions/Discussions in abstract for negative trials: Discuss the primary outcome - if the trial is negative (primary outcome not changed), and the intervention was not used, discuss whether negative results are attributable to lack of uptake and discuss reasons. (Note: Only report in the abstract what the main paper is reporting. If this information is missing from the main body of text, consider adding it)

	1	2	3	4	5	
subitem not at all important	○	○	○	○	○	essential

Does your paper address subitem 1b-v?

Copy and paste relevant sections from the manuscript abstract (include quotes in quotation marks "like this" to indicate direct quotes from your manuscript), or elaborate on this item by providing additional information not in the ms, or briefly explain why the item is not applicable/relevant for your study

Votre réponse

INTRODUCTION

2a) In INTRODUCTION: Scientific background and explanation of rationale

2a-i) Problem and the type of system/solution

Describe the problem and the type of system/solution that is object of the study: intended as stand-alone intervention vs. incorporated in broader health care program? Intended for a particular patient population? Goals of the intervention, e.g., being more cost-effective to other interventions, replace or complement other solutions? (Note: Details about the intervention are provided in “Methods” under 5)

	1	2	3	4	5	
subitem not at all important	○	○	○	○	○	essential

Does your paper address subitem 2a-i? *

Copy and paste relevant sections from the manuscript (include quotes in quotation marks "like this" to indicate direct quotes from your manuscript), or elaborate on this item by providing additional information not in the ms, or briefly explain why the item is not applicable/relevant for your study

"Mobile health (mHealth) technologies offer accessible, personalized, and cost-effective support. Self-management apps provide education, monitoring, and behavioral reinforcement outside clinical settings."

"This randomized controlled trial evaluates the impact of Cardio-Meds, an mHealth app developed by the Cardiology Department at Geneva University Hospitals (HUG) [22], on HF knowledge and therapeutic adherence in patients with HFrEF or HFmrEF. The study aims to determine whether a mobile intervention can enhance disease understanding and support adherence in a real-world outpatient setting."

2a-ii) Scientific background, rationale: What is known about the (type of) system

Scientific background, rationale: What is known about the (type of) system that is the object of the study (be sure to discuss the use of similar systems for other conditions/diagnoses, if appropiate), motivation for the study, i.e. what are the reasons for and what is the context for this specific study, from which stakeholder viewpoint is the study performed, potential impact of findings [2]. Briefly justify the choice of the comparator.

	1	2	3	4	5	
subitem not at all important	○	○	○	○	○	essential

Does your paper address subitem 2a-ii? *

Copy and paste relevant sections from the manuscript (include quotes in quotation marks "like this" to indicate direct quotes from your manuscript), or elaborate on this item by providing additional information not in the ms, or briefly explain why the item is not applicable/relevant for your study

“Mobile health (mHealth) technologies offer accessible, personalized, and cost-effective support. Self-management apps provide education, monitoring, and behavioral reinforcement outside clinical settings.”
“Recent randomized controlled trials have evaluated multi-component mHealth interventions integrating mobile apps and connected devices, reporting improvements in self-care behaviors and symptom outcomes in HF populations.”
“However, current HF apps often lack validated content and integration into national healthcare systems.”

2b) In INTRODUCTION: Specific objectives or hypotheses

Does your paper address CONSORT subitem 2b? *

Copy and paste relevant sections from the manuscript (include quotes in quotation marks "like this" to indicate direct quotes from your manuscript), or elaborate on this item by providing additional information not in the ms, or briefly explain why the item is not applicable/relevant for your study

"This randomized controlled trial evaluates the impact of Cardio-Meds, an mHealth app developed by the Cardiology Department at Geneva University Hospitals (HUG) [22], on HF knowledge and therapeutic adherence in patients with HFrEF or HFmrEF. The study aims to determine whether a mobile intervention can enhance disease understanding and support adherence in a real-world outpatient setting."

METHODS

3a) Description of trial design (such as parallel, factorial) including allocation ratio

Does your paper address CONSORT subitem 3a? *

Copy and paste relevant sections from the manuscript (include quotes in quotation marks "like this" to indicate direct quotes from your manuscript), or elaborate on this item by providing additional information not in the ms, or briefly explain why the item is not applicable/relevant for your study

"This single-centre, prospective, pilot randomized controlled trial was conducted at HUG. Participants were randomly assigned to either the intervention group (using the Cardio-Meds app) or the control group (usual care)."
"...participants were randomized using a computer-generated random allocation sequence with a 1:1 ratio between the intervention and control groups, without stratification or blocking."

3b) Important changes to methods after trial commencement (such as eligibility criteria), with reasons

Does your paper address CONSORT subitem 3b? *

Copy and paste relevant sections from the manuscript (include quotes in quotation marks "like this" to indicate direct quotes from your manuscript), or elaborate on this item by providing additional information not in the ms, or briefly explain why the item is not applicable/relevant for your study

No important changes to the study methods, eligibility criteria, outcomes, or analyses were made after trial commencement. All procedures and outcomes were predefined prior to participant enrolment.

3b-i) Bug fixes, Downtimes, Content Changes

Bug fixes, Downtimes, Content Changes: ehealth systems are often dynamic systems. A description of changes to methods therefore also includes important changes made on the intervention or comparator during the trial (e.g., major bug fixes or changes in the functionality or content) (5-iii) and other “unexpected events” that may have influenced study design such as staff changes, system failures/downtimes, etc. [2].

	1	2	3	4	5	
subitem not at all important	○	○	○	○	○	essential

Does your paper address subitem 3b-i?

Copy and paste relevant sections from the manuscript (include quotes in quotation marks "like this" to indicate direct quotes from your manuscript), or elaborate on this item by providing additional information not in the ms, or briefly explain why the item is not applicable/relevant for your study

No bug fixes, system downtimes, or content changes were implemented during the trial period. The Cardio-Meds application remained unchanged throughout the study. A single standardized technical support call was provided to some participants to resolve individual installation or functionality issues, without any modification of the intervention content or features.

4a) Eligibility criteria for participants

Does your paper address CONSORT subitem 4a? *

Copy and paste relevant sections from the manuscript (include quotes in quotation marks "like this" to indicate direct quotes from your manuscript), or elaborate on this item by providing additional information not in the ms, or briefly explain why the item is not applicable/relevant for your study

"Participants were eligible for inclusion if they met the following criteria:
- Aged 18 years or older.
- Diagnosed with HF with reduced (≤ 40%, or HFrEF) or mildly reduced (41-49% or HFmrEF) left ventricular ejection fraction (LVEF).
- Currently receiving HF-specific medications.
- No cognitive impairment, and fully capable of decision-making.
- Capability to read, understand, and communicate in French.
- Ownership of a smartphone.
- French speaking.

Participants were excluded if they met any of the following conditions:
- Inability or unwillingness to comply with study procedures.
- Status as an asylum seeker, individuals experiencing homelessness, or detainee"

4a-i) Computer / Internet literacy

Computer / Internet literacy is often an implicit “de facto” eligibility criterion - this should be explicitly clarified.

	1	2	3	4	5	
subitem not at all important	○	○	○	○	○	essential

Does your paper address subitem 4a-i?

Copy and paste relevant sections from the manuscript (include quotes in quotation marks "like this" to indicate direct quotes from your manuscript), or elaborate on this item by providing additional information not in the ms, or briefly explain why the item is not applicable/relevant for your study

Basic computer and Internet literacy was implicitly required through smartphone ownership. No formal assessment of digital literacy was performed.

4a-ii) Open vs. closed, web-based vs. face-to-face assessments:

Open vs. closed, web-based vs. face-to-face assessments: Mention how participants were recruited (online vs. offline), e.g., from an open access website or from a clinic, and clarify if this was a purely web-based trial, or there were face-to-face components (as part of the intervention or for assessment), i.e., to what degree got the study team to know the participant. In online-only trials, clarify if participants were quasi-anonymous and whether having multiple identities was possible or whether technical or logistical measures (e.g., cookies, email confirmation, phone calls) were used to detect/prevent these.

	1	2	3	4	5	
subitem not at all important	○	○	○	○	○	essential

Does your paper address subitem 4a-ii? *

Copy and paste relevant sections from the manuscript (include quotes in quotation marks "like this" to indicate direct quotes from your manuscript), or elaborate on this item by providing additional information not in the ms, or briefly explain why the item is not applicable/relevant for your study

"Participants were recruited from the HF clinic and cardiac rehabilitation programs at HUG from March to November 2024. Two cardiologists and two specialized HF nurses pre-identified eligible participants, who were contacted by a medical student who explained the study and verified smartphone ownership."

The trial was not purely web-based. Baseline and follow-up assessments were conducted via scheduled study sessions and self-administered questionnaires. Intervention group participants installed the Cardio-Meds app on their smartphone, with guidance on usage and for medication entry. A brief standardized mid-study support call was conducted solely to identify and resolve potential technical issues related to app installation or functionality. Participants were known to the study team and were not anonymous.

4a-iii) Information giving during recruitment

Information given during recruitment. Specify how participants were briefed for recruitment and in the informed consent procedures (e.g., publish the informed consent documentation as appendix, see also item X26), as this information may have an effect on user self-selection, user expectation and may also bias results.

	1	2	3	4	5	
subitem not at all important	○	○	○	○	○	essential

Does your paper address subitem 4a-iii?

Copy and paste relevant sections from the manuscript (include quotes in quotation marks "like this" to indicate direct quotes from your manuscript), or elaborate on this item by providing additional information not in the ms, or briefly explain why the item is not applicable/relevant for your study

"...participants [...] were contacted by a medical student who explained the study and verified smartphone ownership. Participation was voluntary, with the option to withdraw at any time without affecting medical care. "

"All participants provided written informed consent prior to participation."

4b) Settings and locations where the data were collected

Does your paper address CONSORT subitem 4b? *

Copy and paste relevant sections from the manuscript (include quotes in quotation marks "like this" to indicate direct quotes from your manuscript), or elaborate on this item by providing additional information not in the ms, or briefly explain why the item is not applicable/relevant for your study

Outcome data were collected using self-administered questionnaires

"The following anonymized usage data were collected from the app:
- Total time spent on the application
- Number of logins
- Time spent on the information section
- number of weight, blood pressure, and heart rate entries over the 30-day follow-up period
- Percentage of correct answers to the quizzes

Clinical and personal data were collected from the electronic medical record at HUG, anonymized, and securely stored on HUG servers for 10 years in compliance with institutional policy."

4b-i) Report if outcomes were (self-)assessed through online questionnaires

Clearly report if outcomes were (self-)assessed through online questionnaires (as common in web-based trials) or otherwise.

	1	2	3	4	5	
subitem not at all important	○	○	○	○	○	essential

Does your paper address subitem 4b-i? *

Copy and paste relevant sections from the manuscript (include quotes in quotation marks "like this" to indicate direct quotes from your manuscript), or elaborate on this item by providing additional information not in the ms, or briefly explain why the item is not applicable/relevant for your study

"Outcome assessment relied on self-administered questionnaires"

4b-ii) Report how institutional affiliations are displayed

Report how institutional affiliations are displayed to potential participants [on ehealth media], as affiliations with prestigious hospitals or universities may affect volunteer rates, use, and reactions with regards to an intervention.(Not a required item – describe only if this may bias results)

	1	2	3	4	5	
subitem not at all important	○	○	○	○	○	essential

Does your paper address subitem 4b-ii?

Copy and paste relevant sections from the manuscript (include quotes in quotation marks "like this" to indicate direct quotes from your manuscript), or elaborate on this item by providing additional information not in the ms, or briefly explain why the item is not applicable/relevant for your study

Participants were contacted directly by the study team through the HF clinic and cardiac rehabilitation programs at HUG. Institutional affiliations were not specifically displayed to participants as part of the recruitment process.

5) The interventions for each group with sufficient details to allow replication, including how and when they were actually administered

5-i) Mention names, credential, affiliations of the developers, sponsors, and owners

Mention names, credential, affiliations of the developers, sponsors, and owners [6] (if authors/evaluators are owners or developer of the software, this needs to be declared in a “Conflict of interest” section or mentioned elsewhere in the manuscript).

	1	2	3	4	5	
subitem not at all important	○	○	○	○	○	essential

Does your paper address subitem 5-i?

Copy and paste relevant sections from the manuscript (include quotes in quotation marks "like this" to indicate direct quotes from your manuscript), or elaborate on this item by providing additional information not in the ms, or briefly explain why the item is not applicable/relevant for your study

"Cardio-Meds is a research version of a mHealth app developed at HUG through collaboration between cardiology, pharmacy, the medical directorate, and IT departments."

5-ii) Describe the history/development process

Describe the history/development process of the application and previous formative evaluations (e.g., focus groups, usability testing), as these will have an impact on adoption/use rates and help with interpreting results.

	1	2	3	4	5	
subitem not at all important	○	○	○	○	○	essential

Does your paper address subitem 5-ii?

Copy and paste relevant sections from the manuscript (include quotes in quotation marks "like this" to indicate direct quotes from your manuscript), or elaborate on this item by providing additional information not in the ms, or briefly explain why the item is not applicable/relevant for your study

"A recent usability study rated it good to excellent (23), supporting its feasibility in the HF population."

5-iii) Revisions and updating

Revisions and updating. Clearly mention the date and/or version number of the application/intervention (and comparator, if applicable) evaluated, or describe whether the intervention underwent major changes during the evaluation process, or whether the development and/or content was “frozen” during the trial. Describe dynamic components such as news feeds or changing content which may have an impact on the replicability of the intervention (for unexpected events see item 3b).

	1	2	3	4	5	
subitem not at all important	○	○	○	○	○	essential

Does your paper address subitem 5-iii?

Copy and paste relevant sections from the manuscript (include quotes in quotation marks "like this" to indicate direct quotes from your manuscript), or elaborate on this item by providing additional information not in the ms, or briefly explain why the item is not applicable/relevant for your study

"Cardio-Meds is a research version of a mHealth app developed at HUG."

This version has been used only for the study to get the data of usage. At the end of the study, both groups could install SwissMeds, the publicly released version of the app, soon to include modules for other chronic conditions.

5-iv) Quality assurance methods

Provide information on quality assurance methods to ensure accuracy and quality of information provided [1], if applicable.

	1	2	3	4	5	
subitem not at all important	○	○	○	○	○	essential

Does your paper address subitem 5-iv?

Copy and paste relevant sections from the manuscript (include quotes in quotation marks "like this" to indicate direct quotes from your manuscript), or elaborate on this item by providing additional information not in the ms, or briefly explain why the item is not applicable/relevant for your study

"The app is designed to ensure ease of use and fulfils legal and best practice requirements for data protection and confidentiality."

"Clinical and personal data were collected from the electronic medical record at HUG, anonymized, and securely stored on HUG servers for 10 years in compliance with institutional policy."

5-v) Ensure replicability by publishing the source code, and/or providing screenshots/screen-capture video, and/or providing flowcharts of the algorithms used

Ensure replicability by publishing the source code, and/or providing screenshots/screen-capture video, and/or providing flowcharts of the algorithms used. Replicability (i.e., other researchers should in principle be able to replicate the study) is a hallmark of scientific reporting.

	1	2	3	4	5	
subitem not at all important	○	○	○	○	○	essential

Does your paper address subitem 5-v?

Copy and paste relevant sections from the manuscript (include quotes in quotation marks "like this" to indicate direct quotes from your manuscript), or elaborate on this item by providing additional information not in the ms, or briefly explain why the item is not applicable/relevant for your study

"Key features include:
• Educational content and daily quizzes on HF, its treatment, and lifestyle. The content is organized into four chapters (My condition, warning signs, my medication and lifestyle, Figure 2). The quizzes provide immediate feedback and brief explanations (Figure 3).
• Tools to track weight, blood pressure, and heart rate, enabling early detection of decompensation. Graphs can be generated and used during consultations (Figure 1).
• A treatment plan, easily accessible on the user's phone, allows patients to enter their medication regimen (Figure 4). Medications can be added by scanning the barcode, enabling the app to identify the drug via access to the Swiss medication database. The user can then specify the dosage and timing for each medication intake (Figure 5).
• Intake confirmation and optional reminders to support adherence (Figures 1, 4, and 6)."

SwissMeds, the publicly released and freely available version of the app, includes similar functionalities and will allow replication of key intervention components.

5-vi) Digital preservation

Digital preservation: Provide the URL of the application, but as the intervention is likely to change or disappear over the course of the years; also make sure the intervention is archived (Internet Archive, webcitation.org, and/or publishing the source code or screenshots/videos alongside the article). As pages behind login screens cannot be archived, consider creating demo pages which are accessible without login.

	1	2	3	4	5	
subitem not at all important	◯	◯	◯	◯	◯	essential

Does your paper address subitem 5-vi?

Copy and paste relevant sections from the manuscript (include quotes in quotation marks "like this" to indicate direct quotes from your manuscript), or elaborate on this item by providing additional information not in the ms, or briefly explain why the item is not applicable/relevant for your study

The research version of Cardio-Meds is not publicly available. Screenshots of the app are provided in the manuscript for replicability. The publicly released app SwissMeds is freely available on App Store (https://www.google.com/url?sa=t&source=web&rct=j&opi=89978449&url=https://apps.apple.com/ch/app/swiss-meds/id1509739957%3Fl%3Den-GB&ved=2ahUKEwinudyf89GRAxUr9wIHHZBHAOYQFnoECCIQAQ&usg=AOvVaw2bL9kxQD3W0T00ThtGKgi4) and on google play (https://play.google.com/store/apps/details?id=ch.hug.swissmeds&hl=fr_CH)

5-vii) Access

Access: Describe how participants accessed the application, in what setting/context, if they had to pay (or were paid) or not, whether they had to be a member of specific group. If known, describe how participants obtained “access to the platform and Internet” [1]. To ensure access for editors/reviewers/readers, consider to provide a “backdoor” login account or demo mode for reviewers/readers to explore the application (also important for archiving purposes, see vi).

	1	2	3	4	5	
subitem not at all important	○	○	○	○	○	essential

Does your paper address subitem 5-vii? *

Copy and paste relevant sections from the manuscript (include quotes in quotation marks "like this" to indicate direct quotes from your manuscript), or elaborate on this item by providing additional information not in the ms, or briefly explain why the item is not applicable/relevant for your study

Participants accessed the Cardio-Meds app on their own smartphones during scheduled study sessions or individually at HUG. No payment was required, and participation was voluntary. Participants were pre-identified through the HF clinic and cardiac rehabilitation programs and had to own a smartphone and be able to use it.

5-viii) Mode of delivery, features/functionalities/components of the intervention and comparator, and the theoretical framework

Describe mode of delivery, features/functionalities/components of the intervention and comparator, and the theoretical framework [6] used to design them (instructional strategy [1], behaviour change techniques, persuasive features, etc., see e.g., [7, 8] for terminology). This includes an in-depth description of the content (including where it is coming from and who developed it) [1],” whether [and how] it is tailored to individual circumstances and allows users to track their progress and receive feedback” [6]. This also includes a description of communication delivery channels and – if computer-mediated communication is a component – whether communication was synchronous or asynchronous [6]. It also includes information on presentation strategies [1], including page design principles, average amount of text on pages, presence of hyperlinks to other resources, etc. [1].

	1	2	3	4	5	
subitem not at all important	○	○	○	○	○	essential

Does your paper address subitem 5-viii? *

Copy and paste relevant sections from the manuscript (include quotes in quotation marks "like this" to indicate direct quotes from your manuscript), or elaborate on this item by providing additional information not in the ms, or briefly explain why the item is not applicable/relevant for your study

"Cardio-Meds is a research version of a mHealth app developed at HUG through collaboration between cardiology, pharmacy, the medical directorate, and IT departments. A recent usability study rated it good to excellent (23), supporting its feasibility in the HF population.
[23]. It is designed with and for patients, to help individuals with HF manage their condition through educational resources, self-monitoring, and adherence support.
Key features include:
• Educational content and daily quizzes on HF, its treatment, and lifestyle. The content is organized into four chapters (My condition, warning signs, my medication and lifestyle, Figure 2). The quizzes provide immediate feedback and brief explanations (Figure 3).
• Tools to track weight, blood pressure, and heart rate, enabling early detection of decompensation. Graphs can be generated and used during consultations (Figure 1).
• A treatment plan, easily accessible on the user's phone, allows patients to enter their medication regimen (Figure 4). Medications can be added by scanning the barcode, enabling the app to identify the drug via access to the Swiss medication database. The user can then specify the dosage and timing for each medication intake (Figure 5).
• Intake confirmation and optional reminders to support adherence (Figures 1, 4, and 6).
Cardio-Meds enables patients to manage health data, access structured learning, and share information with providers. The app is designed to ensure ease of use and fulfils legal and best practice requirements for data protection and confidentiality."

5-ix) Describe use parameters

Describe use parameters (e.g., intended “doses” and optimal timing for use). Clarify what instructions or recommendations were given to the user, e.g., regarding timing, frequency, heaviness of use, if any, or was the intervention used ad libitum.

	1	2	3	4	5	
subitem not at all important	○	○	○	○	○	essential

Does your paper address subitem 5-ix?

Copy and paste relevant sections from the manuscript (include quotes in quotation marks "like this" to indicate direct quotes from your manuscript), or elaborate on this item by providing additional information not in the ms, or briefly explain why the item is not applicable/relevant for your study

No specific usage instructions or mandatory schedule were given. Participants were explained the functionalities of the Cardio-Meds app, including tracking vital signs and weight, completing quizzes, and reviewing educational content. It was highlighted that using the app daily could be beneficial for entering health data and completing quizzes, but participants were free to use it at their own discretion.

5-x) Clarify the level of human involvement

Clarify the level of human involvement (care providers or health professionals, also technical assistance) in the e-intervention or as co-intervention (detail number and expertise of professionals involved, if any, as well as “type of assistance offered, the timing and frequency of the support, how it is initiated, and the medium by which the assistance is delivered”. It may be necessary to distinguish between the level of human involvement required for the trial, and the level of human involvement required for a routine application outside of a RCT setting (discuss under item 21 – generalizability).

	1	2	3	4	5	
subitem not at all important	○	○	○	○	○	essential

Does your paper address subitem 5-x?

Copy and paste relevant sections from the manuscript (include quotes in quotation marks "like this" to indicate direct quotes from your manuscript), or elaborate on this item by providing additional information not in the ms, or briefly explain why the item is not applicable/relevant for your study

"Intervention group participants installed the Cardio-Meds app on their smartphone, with guidance on usage and for medication entry. A brief standardized mid-study support call was conducted solely to identify and resolve potential technical issues related to app installation or functionality."

5-xi) Report any prompts/reminders used

Report any prompts/reminders used: Clarify if there were prompts (letters, emails, phone calls, SMS) to use the application, what triggered them, frequency etc. It may be necessary to distinguish between the level of prompts/reminders required for the trial, and the level of prompts/reminders for a routine application outside of a RCT setting (discuss under item 21 – generalizability).

	1	2	3	4	5	
subitem not at all important	○	○	○	○	○	essential

Does your paper address subitem 5-xi? *

Copy and paste relevant sections from the manuscript (include quotes in quotation marks "like this" to indicate direct quotes from your manuscript), or elaborate on this item by providing additional information not in the ms, or briefly explain why the item is not applicable/relevant for your study

Participants received optional intake reminders through the Cardio-Meds app to support medication adherence. No additional prompts, emails, phone calls, or SMS messages were sent to encourage app usage. A single standardized mid-study support call was conducted only to resolve technical issues with app installation or functionality. Outside of the trial, the app provides similar optional reminders, but no additional human-initiated prompts are required.

5-xii) Describe any co-interventions (incl. training/support)

Describe any co-interventions (incl. training/support): Clearly state any interventions that are provided in addition to the targeted eHealth intervention, as ehealth intervention may not be designed as stand-alone intervention. This includes training sessions and support [1]. It may be necessary to distinguish between the level of training required for the trial, and the level of training for a routine application outside of a RCT setting (discuss under item 21 – generalizability.

	1	2	3	4	5	
subitem not at all important	○	○	○	○	○	essential

Does your paper address subitem 5-xii? *

Copy and paste relevant sections from the manuscript (include quotes in quotation marks "like this" to indicate direct quotes from your manuscript), or elaborate on this item by providing additional information not in the ms, or briefly explain why the item is not applicable/relevant for your study

Intervention group participants received guidance during the first study session on installing and using the Cardio-Meds app, including entering medication regimens. A brief standardized mid-study support call was conducted solely to identify and resolve potential technical issues related to app installation or functionality. No additional medical advice, educational reinforcement, or adherence-related counseling was provided. Outside of the trial, the app is intended to be used independently by patients without routine professional training or support.

6a) Completely defined pre-specified primary and secondary outcome measures, including how and when they were assessed

Does your paper address CONSORT subitem 6a? *

Copy and paste relevant sections from the manuscript (include quotes in quotation marks "like this" to indicate direct quotes from your manuscript), or elaborate on this item by providing additional information not in the ms, or briefly explain why the item is not applicable/relevant for your study

"Primary Outcome:
Heart failure knowledge and self-management were assessed using the Dutch Heart Failure Knowledge Scale (DHFKS), a validated 15-item questionnaire covering general knowledge, dietary recommendations, sodium and fluid restrictions, and recognition of alarm symptoms. This tool was selected for its validity and sensitivity in evaluating the effect of therapeutic education in patients with HF. The questionnaire was translated into French using neural machine translation (DeepL) and reviewed for clinical accuracy and clarity by an experienced heart failure specialist (PM). No formal forward–backward translation or psychometric validation of the French version was performed, and the scale was therefore used as an exploratory measure to assess within-group changes in knowledge rather than as a fully validated French-language instrument. Scores range from 0 to 15, with higher scores indicating better knowledge. Based on pragmatic and clinical considerations, an absolute improvement of 2 points on the DHFKS was prespecified as a meaningful change for sample size estimation. This threshold was used as an assumption for planning purposes rather than as a validated minimal clinically important difference. (Appendix 1).

Secondary Outcome:
Medication adherence was assessed using the Basel Assessment of Adherence to Immunosuppressive Medication Scale (BAASIS®), a structured self-report questionnaire originally validated in transplant populations. In the absence of a HF–specific validated adherence questionnaire at the time of study design, the BAASIS® was used as an exploratory tool to capture core adherence behaviors (initiation, implementation, and persistence) relevant to chronic disease management. BAASIS® covers three dimensions of adherence behavior:

- Initiation (starting newly prescribed medications during the reference period)
- Implementation (missed doses, wrong dosage or timing)
- Persistence (discontinuation without medical advice)

Responses are binary (“yes” / “no”), with frequency details for selected items. For the “Initiation” domain, analyses were restricted to participants who had a newly prescribed medication during the respective reference period, as specified by the BAASIS® instrument; therefore, denominators vary across time points and do not reflect missing data or participant exclusion. A validated French version was used (Appendix 2).

Exploratory Outcome:
Usability of the application was evaluated in the intervention group using the System Usability Scale (SUS), a 10-item questionnaire with responses on a 5-point Likert scale. The final score ranges from 0 to 100, with higher scores reflecting better usability (Appendix 3)."

6a-i) Online questionnaires: describe if they were validated for online use and apply CHERRIES items to describe how the questionnaires were designed/deployed

If outcomes were obtained through online questionnaires, describe if they were validated for online use and apply CHERRIES items to describe how the questionnaires were designed/deployed [9].

	1	2	3	4	5	
subitem not at all important	○	○	○	○	○	essential

Does your paper address subitem 6a-i?

Copy and paste relevant sections from manuscript text

Outcomes were assessed using self-administered questionnaires completed during face-to-face study sessions. No online questionnaires were used.

6a-ii) Describe whether and how “use” (including intensity of use/dosage) was defined/measured/monitored

Describe whether and how “use” (including intensity of use/dosage) was defined/measured/monitored (logins, logfile analysis, etc.). Use/adoption metrics are important process outcomes that should be reported in any ehealth trial.

	1	2	3	4	5	
subitem not at all important	○	○	○	○	○	essential

Does your paper address subitem 6a-ii?

Copy and paste relevant sections from manuscript text

"The following anonymized usage data were collected from the app:
• Total time spent on the application
• Number of logins
• Time spent on the information section
• number of weight, blood pressure, and heart rate entries over the 30-day follow-up period
• Percentage of correct answers to the quizzes"

6a-iii) Describe whether, how, and when qualitative feedback from participants was obtained

Describe whether, how, and when qualitative feedback from participants was obtained (e.g., through emails, feedback forms, interviews, focus groups).

	1	2	3	4	5	
subitem not at all important	○	○	○	○	○	essential

Does your paper address subitem 6a-iii?

Copy and paste relevant sections from manuscript text

"Thirty days later, all participants returned for a second session, [...]. Intervention participants also completed a usability questionnaire and had the possibility to give non-directed feedback on the app."

6b) Any changes to trial outcomes after the trial commenced, with reasons

Does your paper address CONSORT subitem 6b? *

Copy and paste relevant sections from the manuscript (include quotes in quotation marks "like this" to indicate direct quotes from your manuscript), or elaborate on this item by providing additional information not in the ms, or briefly explain why the item is not applicable/relevant for your study

No changes to trial outcomes were made after the trial commenced.

7a) How sample size was determined

NPT: When applicable, details of whether and how the clustering by care provides or centers was addressed

7a-i) Describe whether and how expected attrition was taken into account when calculating the sample size

Describe whether and how expected attrition was taken into account when calculating the sample size.

	1	2	3	4	5	
subitem not at all important	○	○	○	○	○	essential

Does your paper address subitem 7a-i?

Copy and paste relevant sections from manuscript title (include quotes in quotation marks "like this" to indicate direct quotes from your manuscript), or elaborate on this item by providing additional information not in the ms, or briefly explain why the item is not applicable/relevant for your study

"The sample size was estimated to inform feasibility rather than to power a definitive efficacy trial. Based on a mean control group DHFKS score of 10.9 (SD 2.3), an anticipated absolute between-group difference of 2 points, a power of 80%, and a two-sided significance level (α) of 0.05, an initial sample size of approximately 40–45 participants was estimated. Allowing for an anticipated dropout rate of 25–30%, a target sample size of 50 participants (25 per group) was considered feasible and appropriate for this pilot exploratory randomized controlled trial."

7b) When applicable, explanation of any interim analyses and stopping guidelines

Does your paper address CONSORT subitem 7b? *

Copy and paste relevant sections from the manuscript (include quotes in quotation marks "like this" to indicate direct quotes from your manuscript), or elaborate on this item by providing additional information not in the ms, or briefly explain why the item is not applicable/relevant for your study

No interim analyses or stopping guidelines were planned or implemented for this pilot trial.

8a) Method used to generate the random allocation sequence

NPT: When applicable, how care providers were allocated to each trial group

Does your paper address CONSORT subitem 8a? *

Copy and paste relevant sections from the manuscript (include quotes in quotation marks "like this" to indicate direct quotes from your manuscript), or elaborate on this item by providing additional information not in the ms, or briefly explain why the item is not applicable/relevant for your study

"After providing oral consent and before the first session, participants were randomized using a computer-generated random allocation sequence with a 1:1 ratio between the intervention and control groups, without stratification or blocking. The allocation sequence was generated by a member of the research team not involved in participant recruitment or outcome assessment. Randomization was conducted prior to the first study session for logistical reasons related to group-based study organization."

8b) Type of randomisation; details of any restriction (such as blocking and block size)

Does your paper address CONSORT subitem 8b? *

Copy and paste relevant sections from the manuscript (include quotes in quotation marks "like this" to indicate direct quotes from your manuscript), or elaborate on this item by providing additional information not in the ms, or briefly explain why the item is not applicable/relevant for your study

"After providing oral consent and before the first session, participants were randomized using a computer-generated random allocation sequence with a 1:1 ratio between the intervention and control groups, without stratification or blocking."

9) Mechanism used to implement the random allocation sequence (such as sequentially numbered containers), describing any steps taken to conceal the sequence until interventions were assigned

Does your paper address CONSORT subitem 9? *

Copy and paste relevant sections from the manuscript (include quotes in quotation marks "like this" to indicate direct quotes from your manuscript), or elaborate on this item by providing additional information not in the ms, or briefly explain why the item is not applicable/relevant for your study

"The allocation sequence was generated by a member of the research team not involved in participant recruitment or outcome assessment. Randomization was conducted prior to the first study session for logistical reasons related to group-based study organization. Participants were enrolled by study investigators, and baseline questionnaires were administered before any information regarding group allocation was disclosed. Neither participants nor study personnel responsible for distributing and collecting the questionnaires were aware of group assignment at the time of baseline data collection."

10) Who generated the random allocation sequence, who enrolled participants, and who assigned participants to interventions

Does your paper address CONSORT subitem 10? *

Copy and paste relevant sections from the manuscript (include quotes in quotation marks "like this" to indicate direct quotes from your manuscript), or elaborate on this item by providing additional information not in the ms, or briefly explain why the item is not applicable/relevant for your study

"The allocation sequence was generated by a member of the research team not involved in participant recruitment or outcome assessment. [...]Participants were enrolled by study investigators,..."

11a) If done, who was blinded after assignment to interventions (for example, participants, care providers, those assessing outcomes) and how

NPT: Whether or not administering co-interventions were blinded to group assignment

11a-i) Specify who was blinded, and who wasn’t

Specify who was blinded, and who wasn’t. Usually, in web-based trials it is not possible to blind the participants [1, 3] (this should be clearly acknowledged), but it may be possible to blind outcome assessors, those doing data analysis or those administering co-interventions (if any).

	1	2	3	4	5	
subitem not at all important	○	○	○	○	○	essential

Does your paper address subitem 11a-i? *

Copy and paste relevant sections from the manuscript (include quotes in quotation marks "like this" to indicate direct quotes from your manuscript), or elaborate on this item by providing additional information not in the ms, or briefly explain why the item is not applicable/relevant for your study

"Given the nature of the digital intervention, blinding of participants and study staff after allocation was not feasible. Outcome assessment relied on self-administered questionnaires, and data analysis was conducted on de-identified datasets, with analysts not involved in intervention delivery."

11a-ii) Discuss e.g., whether participants knew which intervention was the “intervention of interest” and which one was the “comparator”

Informed consent procedures (4a-ii) can create biases and certain expectations - discuss e.g., whether participants knew which intervention was the “intervention of interest” and which one was the “comparator”.

	1	2	3	4	5	
subitem not at all important	○	○	○	○	○	essential

Does your paper address subitem 11a-ii?

Copy and paste relevant sections from the manuscript (include quotes in quotation marks "like this" to indicate direct quotes from your manuscript), or elaborate on this item by providing additional information not in the ms, or briefly explain why the item is not applicable/relevant for your study

Participants were informed that the study involved two groups, but they were aware that the Cardio-Meds app represented the active intervention and that the control group would receive usual care. This was explained during the informed consent process. Awareness of group assignment may have influenced expectations, particularly regarding the app's potential benefits, but baseline assessments were completed before revealing group allocation to minimize bias.

11b) If relevant, description of the similarity of interventions

(this item is usually not relevant for ehealth trials as it refers to similarity of a placebo or sham intervention to a active medication/intervention)

Does your paper address CONSORT subitem 11b? *

Copy and paste relevant sections from the manuscript (include quotes in quotation marks "like this" to indicate direct quotes from your manuscript), or elaborate on this item by providing additional information not in the ms, or briefly explain why the item is not applicable/relevant for your study

Not applicable. The control group received usual care and no placebo intervention was provided.

12a) Statistical methods used to compare groups for primary and secondary outcomes

NPT: When applicable, details of whether and how the clustering by care providers or centers was addressed

Does your paper address CONSORT subitem 12a? *

Copy and paste relevant sections from the manuscript (include quotes in quotation marks "like this" to indicate direct quotes from your manuscript), or elaborate on this item by providing additional information not in the ms, or briefly explain why the item is not applicable/relevant for your study

"Descriptive statistics were used to summarize baseline characteristics and outcomes. Continuous variables are presented as mean ± standard deviation (SD) and were compared between groups using Student's t-test when appropriate. Categorical variables are presented as frequencies and percentages and were compared using Chi-square or Fisher's exact tests, depending on expected cell counts.
The primary outcome, change in heart failure knowledge, was analysed using an analysis of covariance (ANCOVA), with the post-intervention Dutch Heart Failure Knowledge Scale (DHFKS) score as the dependent variable, study group as the independent variable, and baseline DHFKS score as a covariate. No post-hoc adjustment was performed for other baseline variables, given the limited sample size and exploratory nature of this pilot study."

12a-i) Imputation techniques to deal with attrition / missing values

Imputation techniques to deal with attrition / missing values: Not all participants will use the intervention/comparator as intended and attrition is typically high in ehealth trials. Specify how participants who did not use the application or dropped out from the trial were treated in the statistical analysis (a complete case analysis is strongly discouraged, and simple imputation techniques such as LOCF may also be problematic [4]).

	1	2	3	4	5	
subitem not at all important	○	○	○	○	○	essential

Does your paper address subitem 12a-i? *

Copy and paste relevant sections from the manuscript (include quotes in quotation marks "like this" to indicate direct quotes from your manuscript), or elaborate on this item by providing additional information not in the ms, or briefly explain why the item is not applicable/relevant for your study

"Participants with missing follow-up data were not included in the corresponding outcome analyses. No participants were excluded post-randomization due to intervention-related reasons; analyses were restricted to participants with available baseline and follow-up data for the corresponding outcomes."

12b) Methods for additional analyses, such as subgroup analyses and adjusted analyses

Does your paper address CONSORT subitem 12b? *

Copy and paste relevant sections from the manuscript (include quotes in quotation marks "like this" to indicate direct quotes from your manuscript), or elaborate on this item by providing additional information not in the ms, or briefly explain why the item is not applicable/relevant for your study

"No post-hoc adjustment was performed for other baseline variables, given the limited sample size and exploratory nature of this pilot study."

X26) REB/IRB Approval and Ethical Considerations [recommended as subheading under "Methods"] (not a CONSORT item)

X26-i) Comment on ethics committee approval

	1	2	3	4	5	
subitem not at all important	○	○	○	○	○	essential

Does your paper address subitem X26-i?

Copy and paste relevant sections from the manuscript (include quotes in quotation marks "like this" to indicate direct quotes from your manuscript), or elaborate on this item by providing additional information not in the ms, or briefly explain why the item is not applicable/relevant for your study

"The study was submitted to the local ethics committee (Commission cantonale d'éthique de la recherche, CCER, Geneva, Switzerland; ID 2023-01337) for review. The CCER reviewed the submission (ID 2023-01337) and determined that formal approval was not required given the low-risk, educational nature of the study and the absence of clinical or safety endpoints. All participants provided written informed consent prior to participation."

x26-ii) Outline informed consent procedures

Outline informed consent procedures e.g., if consent was obtained offline or online (how? Checkbox, etc.?), and what information was provided (see 4a-ii). See [6] for some items to be included in informed consent documents.

	1	2	3	4	5	
subitem not at all important	○	○	○	○	○	essential

Does your paper address subitem X26-ii?

Copy and paste relevant sections from the manuscript (include quotes in quotation marks "like this" to indicate direct quotes from your manuscript), or elaborate on this item by providing additional information not in the ms, or briefly explain why the item is not applicable/relevant for your study

Written informed consent was obtained from all participants during face-to-face study sessions before any study procedures were initiated. Participants were provided with information about the study objectives, procedures, voluntary participation, and the option to withdraw at any time without affecting their medical care. Study staff explained the functionalities of the Cardio-Meds app and the nature of the intervention and control groups, ensuring participants understood what participation entailed.

X26-iii) Safety and security procedures

Safety and security procedures, incl. privacy considerations, and any steps taken to reduce the likelihood or detection of harm (e.g., education and training, availability of a hotline)

	1	2	3	4	5	
subitem not at all important	○	○	○	○	○	essential

Does your paper address subitem X26-iii?

Copy and paste relevant sections from the manuscript (include quotes in quotation marks "like this" to indicate direct quotes from your manuscript), or elaborate on this item by providing additional information not in the ms, or briefly explain why the item is not applicable/relevant for your study

"Clinical and personal data were collected from the electronic medical record at HUG, anonymized, and securely stored on HUG servers for 10 years in compliance with institutional policy."

"The app is designed to ensure ease of use and fulfils legal and best practice requirements for data protection and confidentiality."

RESULTS

13a) For each group, the numbers of participants who were randomly assigned, received intended treatment, and were analysed for the primary outcome
NPT: The number of care providers or centers performing the intervention in each group and the number of patients treated by each care provider in each center

Does your paper address CONSORT subitem 13a? *

Copy and paste relevant sections from the manuscript (include quotes in quotation marks "like this" to indicate direct quotes from your manuscript), or elaborate on this item by providing additional information not in the ms, or briefly explain why the item is not applicable/relevant for your study

"Among the 61 participants, 31 were randomly assigned to the intervention group and 30 to the control group. Before the first session, 11 participants dropped out: 10 did not attend the scheduled session, and one participant in the intervention group underwent heart transplantation. Ultimately, 50 participants attended the first session and 49 were included, as 1 participant did not own a smartphone."

13b) For each group, losses and exclusions after randomisation, together with reasons

Does your paper address CONSORT subitem 13b? (NOTE: Preferably, this is shown in a CONSORT flow diagram) *

Copy and paste relevant sections from the manuscript (include quotes in quotation marks "like this" to indicate direct quotes from your manuscript), or elaborate on this item by providing additional information not in the ms, or briefly explain why the item is not applicable/relevant for your study

"During the study, one participant in the control group underwent a heart transplantation and was therefore unable to participate in the second session and two others did not complete the BAASIS® questionnaire at follow-up."

13b-i) Attrition diagram

Strongly recommended: An attrition diagram (e.g., proportion of participants still logging in or using the intervention/comparator in each group plotted over time, similar to a survival curve) or other figures or tables demonstrating usage/dose/engagement.

	1	2	3	4	5	
subitem not at all important	○	○	○	○	○	essential

Does your paper address subitem 13b-i?

Copy and paste relevant sections from the manuscript or cite the figure number if applicable (include quotes in quotation marks "like this" to indicate direct quotes from your manuscript), or elaborate on this item by providing additional information not in the ms, or briefly explain why the item is not applicable/relevant for your study

Participant flow and attrition are illustrated in Figure 8. The diagram shows the number of participants randomly assigned, those who attended the first session, and those who completed follow-up assessments in each group.

14a) Dates defining the periods of recruitment and follow-up

Does your paper address CONSORT subitem 14a? *

Copy and paste relevant sections from the manuscript (include quotes in quotation marks "like this" to indicate direct quotes from your manuscript), or elaborate on this item by providing additional information not in the ms, or briefly explain why the item is not applicable/relevant for your study

"Participants were recruited from the HF clinic and cardiac rehabilitation programs at HUG from March to November 2024."

14a-i) Indicate if critical “secular events” fell into the study period

Indicate if critical “secular events” fell into the study period, e.g., significant changes in Internet resources available or “changes in computer hardware or Internet delivery resources”

	1	2	3	4	5	
subitem not at all important	○	○	○	○	○	essential

Does your paper address subitem 14a-i?

Copy and paste relevant sections from the manuscript (include quotes in quotation marks "like this" to indicate direct quotes from your manuscript), or elaborate on this item by providing additional information not in the ms, or briefly explain why the item is not applicable/relevant for your study

No critical secular events, such as changes in Internet resources, app availability, or hardware/software infrastructure, occurred during the study period.

14b) Why the trial ended or was stopped (early)

Does your paper address CONSORT subitem 14b? *

Copy and paste relevant sections from the manuscript (include quotes in quotation marks "like this" to indicate direct quotes from your manuscript), or elaborate on this item by providing additional information not in the ms, or briefly explain why the item is not applicable/relevant for your study

The trial was completed as planned and was not stopped early.

15) A table showing baseline demographic and clinical characteristics for each group

NPT: When applicable, a description of care providers (case volume, qualification, expertise, etc.) and centers (volume) in each group

Does your paper address CONSORT subitem 15? *

Copy and paste relevant sections from the manuscript (include quotes in quotation marks "like this" to indicate direct quotes from your manuscript), or elaborate on this item by providing additional information not in the ms, or briefly explain why the item is not applicable/relevant for your study

Baseline demographic and clinical characteristics of participants in each group are presented in Table 1. The table includes age, sex, language, HF etiology, LVEF, comorbidities, and medication use, providing a comprehensive overview of participant characteristics at study entry.

15-i) Report demographics associated with digital divide issues

In ehealth trials it is particularly important to report demographics associated with digital divide issues, such as age, education, gender, social-economic status, computer/Internet/ehealth literacy of the participants, if known.

	1	2	3	4	5	
subitem not at all important	○	○	○	○	○	essential

Does your paper address subitem 15-i? *

Copy and paste relevant sections from the manuscript (include quotes in quotation marks "like this" to indicate direct quotes from your manuscript), or elaborate on this item by providing additional information not in the ms, or briefly explain why the item is not applicable/relevant for your study

"This pilot study included a total of 49 adult participants with HF most of whom were male (78%), with a mean age of 62 ± 11.4 years. The majority were French- or Italian-speaking and demonstrated a good level of comprehension of French."

All participants owned a smartphone, ensuring basic digital literacy required for using the Cardio-Meds app.

16) For each group, number of participants (denominator) included in each analysis and whether the analysis was by original assigned groups

16-i) Report multiple "denominators" and provide definitions

Report multiple "denominators" and provide definitions: Report N's (and effect sizes) "across a range of study participation [and use] thresholds" [1], e.g., N exposed, N consented, N used more than x times, N used more than y weeks, N participants "used" the intervention/comparator at specific pre-defined time points of interest (in absolute and relative numbers per group). Always clearly define "use" of the intervention.

	1	2	3	4	5	
subitem not at all important	○	○	○	○	○	essential

Does your paper address subitem 16-i? *

Copy and paste relevant sections from the manuscript (include quotes in quotation marks "like this" to indicate direct quotes from your manuscript), or elaborate on this item by providing additional information not in the ms, or briefly explain why the item is not applicable/relevant for your study

"During the 30-day follow-up period, participants in the intervention group used the application for a mean total duration of 177,4 ± 130.4 minutes, with a mean of 48.6 ± 36.2 logins. Application usage was highly variable across participants. Total usage time had a median of 123 minutes (IQR 74–273), and the number of logins had a median of 43 (IQR 19–85), indicating a skewed distribution with a subset of highly active users. The mean time spent per login was 5.3 ± 3.6 minutes. Participants spent an average of 7.9 minutes on the information section. Weight was recorded an average of 5.9 times, blood pressure and heart rate were entered 6.8 times, corresponding to an average of approximately one entry every 4–5 days. Users who accessed the quiz feature, answered an average of 16.4 questions, with 12.5 correct answers, corresponding to a mean accuracy rate of 0.7. "

16-ii) Primary analysis should be intent-to-treat

Primary analysis should be intent-to-treat, secondary analyses could include comparing only “users”, with the appropriate caveats that this is no longer a randomized sample (see 18-i).

	1	2	3	4	5	
subitem not at all important	○	○	○	○	○	essential

Does your paper address subitem 16-ii?

Copy and paste relevant sections from the manuscript (include quotes in quotation marks "like this" to indicate direct quotes from your manuscript), or elaborate on this item by providing additional information not in the ms, or briefly explain why the item is not applicable/relevant for your study

The primary analysis was conducted according to original randomized group assignment (modified intention-to-treat), including all participants with available baseline and follow-up data. Secondary analyses exploring usage intensity were descriptive.

17a) For each primary and secondary outcome, results for each group, and the estimated effect size and its precision (such as 95% confidence interval)

Does your paper address CONSORT subitem 17a? *

Copy and paste relevant sections from the manuscript (include quotes in quotation marks "like this" to indicate direct quotes from your manuscript), or elaborate on this item by providing additional information not in the ms, or briefly explain why the item is not applicable/relevant for your study

"At baseline (session 1), the mean score was 11.1 ± 2.4 in the intervention group and 10.5 ± 2.9 in the control group. In the primary analysis using ANCOVA adjusted for baseline DHFKS score, the intervention group had significantly higher follow-up knowledge scores than the control group, with an adjusted mean between-group difference of +1.3 points (95% CI [0.40–2.20], p < 0.001). For descriptive purposes, within-group analyses showed that DHFKS scores remained stable in the control group (10.5 ± 2.9 at baseline vs 10.4 ± 3.0 at follow-up; p = 0.82), whereas the intervention group demonstrated a statistically significant increase (11.1 ± 2.4 to 12.4 ± 2.4; p < 0.001)."

"For initiation, the intervention group maintained a score of 100% at both time points, while the control group showed a decrease from 100% at baseline to 80% at one month, corresponding to one out of 5 participants who had not started a newly prescribed medication. The comparison between groups yielded a p-value of 0.382, indicating no statistically significant difference" "Regarding implementation, the intervention group showed a decrease from 64% at baseline to 52% at one month. In contrast, the control group showed a slight increase, from 46% to 48%, likely due to missing responses from three participants at follow-up. The between-group difference was not significant (p = 0.608). For persistence, the intervention group decreased slightly from 100% to 96% after the intervention, while the control group remained relatively stable, from 96% to 95%."

"The mean SUS score in the intervention group was 84.3 ± 15.0 (Appendix 5), indicating excellent usability, as it falls within the 90th percentile of usability scores"

17a-i) Presentation of process outcomes such as metrics of use and intensity of use

In addition to primary/secondary (clinical) outcomes, the presentation of process outcomes such as metrics of use and intensity of use (dose, exposure) and their operational definitions is critical. This does not only refer to metrics of attrition (13-b) (often a binary variable), but also to more continuous exposure metrics such as “average session length”. These must be accompanied by a technical description how a metric like a “session” is defined (e.g., timeout after idle time) [1] (report under item 6a).

	1	2	3	4	5	
subitem not at all important	○	○	○	○	○	essential

Does your paper address subitem 17a-i?

Copy and paste relevant sections from the manuscript (include quotes in quotation marks "like this" to indicate direct quotes from your manuscript), or elaborate on this item by providing additional information not in the ms, or briefly explain why the item is not applicable/relevant for your study

"During the 30-day follow-up period, participants in the intervention group used the application for a mean total duration of 177,4 ± 130.4 minutes, with a mean of 48.6 ± 36.2 logins. Application usage was highly variable across participants. Total usage time had a median of 123 minutes (IQR 74–273), and the number of logins had a median of 43 (IQR 19–85), indicating a skewed distribution with a subset of highly active users. The mean time spent per login was 5.3 ± 3.6 minutes. Participants spent an average of 7.9 minutes on the information section. Weight was recorded an average of 5.9 times, blood pressure and heart rate were entered 6.8 times, corresponding to an average of approximately one entry every 4–5 days. Users who accessed the quiz feature, answered an average of 16.4 questions, with 12.5 correct answers, corresponding to a mean accuracy rate of 0.7."

17b) For binary outcomes, presentation of both absolute and relative effect sizes is recommended

Does your paper address CONSORT subitem 17b? *

Copy and paste relevant sections from the manuscript (include quotes in quotation marks "like this" to indicate direct quotes from your manuscript), or elaborate on this item by providing additional information not in the ms, or briefly explain why the item is not applicable/relevant for your study

"For initiation, the intervention group maintained a score of 100% at both time points, while the control group showed a decrease from 100% at baseline to 80% at one month, corresponding to one out of 5 participants who had not started a newly prescribed medication."
"Regarding implementation, the intervention group showed a decrease from 64% at baseline to 52% at one month. In contrast, the control group showed a slight increase, from 46% to 48%, likely due to missing responses from three participants at follow-up."
"For persistence, the intervention group decreased slightly from 100% to 96% after the intervention, while the control group remained relatively stable, from 96% to 95%. These small variations correspond to one participant in each group: in the control group, one answered "yes" at both time points; in the intervention group, one answered "yes" only at the second session."

18) Results of any other analyses performed, including subgroup analyses and adjusted analyses, distinguishing pre-specified from exploratory

Does your paper address CONSORT subitem 18? *

Copy and paste relevant sections from the manuscript (include quotes in quotation marks "like this" to indicate direct quotes from your manuscript), or elaborate on this item by providing additional information not in the ms, or briefly explain why the item is not applicable/relevant for your study

Exploratory analyses examined associations between application usage metrics (total time, number of logins) and change in DHFKS scores. No significant associations were observed. No pre-specified subgroup or adjusted analyses were conducted.

18-i) Subgroup analysis of comparing only users

A subgroup analysis of comparing only users is not uncommon in ehealth trials, but if done, it must be stressed that this is a self-selected sample and no longer an unbiased sample from a randomized trial (see 16-iii).

	1	2	3	4	5	
subitem not at all important	○	○	○	○	○	essential

Does your paper address subitem 18-i?

Copy and paste relevant sections from the manuscript (include quotes in quotation marks "like this" to indicate direct quotes from your manuscript), or elaborate on this item by providing additional information not in the ms, or briefly explain why the item is not applicable/relevant for your study

No subgroup analyses comparing only users were performed.

19) All important harms or unintended effects in each group
(for specific guidance see CONSORT for harms)

Does your paper address CONSORT subitem 19? *

Copy and paste relevant sections from the manuscript (include quotes in quotation marks "like this" to indicate direct quotes from your manuscript), or elaborate on this item by providing additional information not in the ms, or briefly explain why the item is not applicable/relevant for your study

No important harms or unintended effects related to the intervention or control conditions were observed in any group during the study period.

19-i) Include privacy breaches, technical problems

Include privacy breaches, technical problems. This does not only include physical “harm” to participants, but also incidents such as perceived or real privacy breaches [1], technical problems, and other unexpected/unintended incidents. “Unintended effects” also includes unintended positive effects [2].

	1	2	3	4	5	
subitem not at all important	○	○	○	○	○	essential

Does your paper address subitem 19-i?

Copy and paste relevant sections from the manuscript (include quotes in quotation marks "like this" to indicate direct quotes from your manuscript), or elaborate on this item by providing additional information not in the ms, or briefly explain why the item is not applicable/relevant for your study

No privacy breaches or technical problems occurred during the study. Participants did not report any unintended negative effects, and some positive experiences were noted, such as increased engagement with self-monitoring and educational content.

19-ii) Include qualitative feedback from participants or observations from staff/researchers

Include qualitative feedback from participants or observations from staff/researchers, if available, on strengths and shortcomings of the application, especially if they point to unintended/unexpected effects or uses. This includes (if available) reasons for why people did or did not use the application as intended by the developers.

	1	2	3	4	5	
subitem not at all important	○	○	○	○	○	essential

Does your paper address subitem 19-ii?

Copy and paste relevant sections from the manuscript (include quotes in quotation marks "like this" to indicate direct quotes from your manuscript), or elaborate on this item by providing additional information not in the ms, or briefly explain why the item is not applicable/relevant for your study

"Participants could leave comments at the end of the questionnaires. Overall, the app was seen as well-designed and pleasant to use, with one participant describing it as a "companion that sets the rhythm of his day." Users appreciated features such as the motivational message “Everything is fine today”, medication images for easy identification, and the reminder function. Quizzes and educational content were well received. Reported difficulties included deleting medications after input errors, handling missed doses, or grouping medications, often due to unfamiliarity with the app’s functions. Suggestions included improving text contrast, syncing reminders with smartwatches, managing time zones, integrating with health platforms, storing prescriptions, and adding tracking for weight and blood pressure. Some also requested videos and content on disease progression. These comments will inform future app development."

DISCUSSION

22) Interpretation consistent with results, balancing benefits and harms, and considering other relevant evidence

NPT: In addition, take into account the choice of the comparator, lack of or partial blinding, and unequal expertise of care providers or centers in each group

22-i) Restate study questions and summarize the answers suggested by the data, starting with primary outcomes and process outcomes (use)

Restate study questions and summarize the answers suggested by the data, starting with primary outcomes and process outcomes (use).

	1	2	3	4	5	
subitem not at all important	○	○	○	○	○	essential

Does your paper address subitem 22-i? *

Copy and paste relevant sections from the manuscript (include quotes in quotation marks "like this" to indicate direct quotes from your manuscript), or elaborate on this item by providing additional information not in the ms, or briefly explain why the item is not applicable/relevant for your study

"In this pilot randomized controlled trial conducted in a stable ambulatory HF population, a 30-day intervention using the Cardio-Meds mobile application was associated with a statistically significant short-term improvement in HF knowledge and self-management compared with usual care. After adjustment for baseline knowledge, the intervention group demonstrated a statistically significant between-group improvement of 1.3 points in DHFKS score compared with the control group. The magnitude of improvement, while statistically significant, was smaller than the prespecified effect size and should therefore be interpreted as an exploratory signal rather than a clinically definitive change. In contrast, no significant differences were observed between groups in self-reported medication adherence across the BAASIS® domains; small numerical variations should be interpreted cautiously in light of the pilot design and reliance on self-reported measures. The application demonstrated high usability and acceptability, with excellent System Usability Scale scores and a majority of participants reporting willingness to continue using the app. Taken together, these findings should be interpreted as short-term, exploratory results primarily informing feasibility, usability, and educational impact rather than durable behavioral change."

22-ii) Highlight unanswered new questions, suggest future research

Highlight unanswered new questions, suggest future research.

	1	2	3	4	5	
subitem not at all important	○	○	○	○	○	essential

Does your paper address subitem 22-ii?

Copy and paste relevant sections from the manuscript (include quotes in quotation marks "like this" to indicate direct quotes from your manuscript), or elaborate on this item by providing additional information not in the ms, or briefly explain why the item is not applicable/relevant for your study

"Future research should evaluate Cardio-Meds in larger, multicentre randomized trials with longer follow-up and inclusion of clinical endpoints such as hospitalization, emergency visits, or quality of life. Studying the intervention in the early post-discharge period, when patients are particularly vulnerable and educational needs are high, may be especially informative. Further development could also include adaptation to different literacy levels and languages to enhance accessibility and equity of care."

20) Trial limitations, addressing sources of potential bias, imprecision, and, if relevant, multiplicity of analyses

20-i) Typical limitations in ehealth trials

Typical limitations in ehealth trials: Participants in ehealth trials are rarely blinded. Ehealth trials often look at a multiplicity of outcomes, increasing risk for a Type I error. Discuss biases due to non-use of the intervention/usability issues, biases through informed consent procedures, unexpected events.

	1	2	3	4	5	
subitem not at all important	○	○	○	○	○	essential

Does your paper address subitem 20-i? *

Copy and paste relevant sections from the manuscript (include quotes in quotation marks "like this" to indicate direct quotes from your manuscript), or elaborate on this item by providing additional information not in the ms, or briefly explain why the item is not applicable/relevant for your study

"Several limitations should be acknowledged. First, this pilot study was powered exclusively for the primary outcome of heart failure knowledge. Consequently, it was underpowered to detect differences in secondary outcomes such as medication adherence, particularly in a stable ambulatory population with high baseline adherence. Adherence findings should therefore be interpreted as exploratory and hypothesis-generating rather than confirmatory. Second, the short follow-up period limited assessment of longer-term knowledge retention, sustained behavioral change, and clinical outcomes. Short-term improvements may partly reflect transient or “honeymoon” effects, and longer follow-up will be required to evaluate durability of effects on self-management and adherence.
Third, medication adherence was assessed using self-reported measures, which are inherently subject to recall and social desirability bias. Although validated instruments were used, future studies would benefit from incorporating objective adherence measures such as pharmacy refill data or electronic monitoring.
Fourth, the single-centre design and inclusion of a stable, chronically treated outpatient population may limit generalizability, particularly to recently hospitalized or higher-risk patients.
Fifth, heart failure knowledge was assessed using a French translation of the Dutch Heart Failure Knowledge Scale that did not undergo formal linguistic or psychometric validation. While the items are factual and clinically straightforward and the original scale was developed in a population comparable to the Swiss context, this limits interpretation of absolute score values and the strength of conclusions regarding knowledge gains. In addition, the absence of a validated minimal clinically important difference for the DHFKS constrains assessment of the clinical relevance of the observed improvement. Accordingly, knowledge findings should be considered exploratory.
Sixth, application engagement showed marked heterogeneity, with a small number of highly active users and overall limited use of specific features such as physiological self-monitoring. This skewed distribution limits interpretation of average usage statistics and precluded meaningful dose–response analyses. Consistent with this, no clear association was observed between engagement metrics and improvement in knowledge scores. Although a testing effect from repeated questionnaire administration may have contributed to knowledge gains, this would be expected to affect both groups similarly, and the absence of improvement in the control group suggests that testing alone is unlikely to explain the observed between-group difference.
Finally, the intervention group received a brief mid-study technical support call that was not mirrored in the control group. Although this contact was limited to troubleshooting and did not include educational or behavioral guidance, an attention-related or Hawthorne effect cannot be fully excluded. In addition, baseline imbalances were observed between groups, with the intervention group being younger and including more non-native French speakers. Such differences may occur by chance in small pilot studies and could have influenced engagement, as age may proxy digital literacy. Given the limited sample size, post-hoc adjustment was not performed, and these factors should be considered potential confounders."

21) Generalisability (external validity, applicability) of the trial findings

NPT: External validity of the trial findings according to the intervention, comparators, patients, and care providers or centers involved in the trial

21-i) Generalizability to other populations

Generalizability to other populations: In particular, discuss generalizability to a general Internet population, outside of a RCT setting, and general patient population, including applicability of the study results for other organizations

	1	2	3	4	5	
subitem not at all important	○	○	○	○	○	essential

Does your paper address subitem 21-i?

Copy and paste relevant sections from the manuscript (include quotes in quotation marks "like this" to indicate direct quotes from your manuscript), or elaborate on this item by providing additional information not in the ms, or briefly explain why the item is not applicable/relevant for your study

"Fourth, the single-centre design and inclusion of a stable, chronically treated outpatient population may limit generalizability, particularly to recently hospitalized or higher-risk patients."
"Studying the intervention in the early post-discharge period, when patients are particularly vulnerable and educational needs are high, may be especially informative. "

21-ii) Discuss if there were elements in the RCT that would be different in a routine application setting

Discuss if there were elements in the RCT that would be different in a routine application setting (e.g., prompts/reminders, more human involvement, training sessions or other co-interventions) and what impact the omission of these elements could have on use, adoption, or outcomes if the intervention is applied outside of a RCT setting.

	1	2	3	4	5	
subitem not at all important	○	○	○	○	○	essential

Does your paper address subitem 21-ii?

Copy and paste relevant sections from the manuscript (include quotes in quotation marks "like this" to indicate direct quotes from your manuscript), or elaborate on this item by providing additional information not in the ms, or briefly explain why the item is not applicable/relevant for your study

"Finally, the intervention group received a brief mid-study technical support call that was not mirrored in the control group. Although this contact was limited to troubleshooting and did not include educational or behavioral guidance, an attention-related or Hawthorne effect cannot be fully excluded."
"These findings should be interpreted as exploratory and hypothesis-generating and support further evaluation of Cardio-Meds in larger studies with longer follow-up and clinically relevant outcomes."

OTHER INFORMATION

23) Registration number and name of trial registry

Does your paper address CONSORT subitem 23? *

Copy and paste relevant sections from the manuscript (include quotes in quotation marks "like this" to indicate direct quotes from your manuscript), or elaborate on this item by providing additional information not in the ms, or briefly explain why the item is not applicable/relevant for your study

"This exploratory, single-centre randomized controlled trial was not prospectively registered in a public trial registry. At the time of study initiation, prospective registration was not requested for this type of low-risk, educational intervention. All study outcomes, analyses, and endpoints were predefined prior to data collection and are reported consistently, with no outcome switching. Future larger-scale or multicentre randomized trials evaluating Cardio-Meds will be prospectively registered in accordance with international recommendations."

24) Where the full trial protocol can be accessed, if available

Does your paper address CONSORT subitem 24? *

Cite a Multimedia Appendix, other reference, or copy and paste relevant sections from the manuscript (include quotes in quotation marks "like this" to indicate direct quotes from your manuscript), or elaborate on this item by providing additional information not in the ms, or briefly explain why the item is not applicable/relevant for your study

The full trial protocol is not publicly available. The study was conducted as an exploratory, single-centre pilot randomized controlled trial, and no standalone protocol was published or registered. All key methodological elements, outcomes, and analyses were predefined prior to data collection and are fully described in the Methods section of the manuscript.

25) Sources of funding and other support (such as supply of drugs), role of funders

Does your paper address CONSORT subitem 25? *

Copy and paste relevant sections from the manuscript (include quotes in quotation marks "like this" to indicate direct quotes from your manuscript), or elaborate on this item by providing additional information not in the ms, or briefly explain why the item is not applicable/relevant for your study

"This study was supported by the GEcor Foundation (Fondation privée de la cardiologie universitaire à Genève). The funding body had no role in the study design; data collection, analysis, or interpretation; manuscript preparation; or the decision to submit the manuscript for publication."

X27) Conflicts of Interest (not a CONSORT item)

X27-i) State the relation of the study team towards the system being evaluated

In addition to the usual declaration of interests (financial or otherwise), also state the relation of the study team towards the system being evaluated, i.e., state if the authors/evaluators are distinct from or identical with the developers/sponsors of the intervention.

	1	2	3	4	5	
subitem not at all important	○	○	○	○	○	essential

Does your paper address subitem X27-i?

Copy and paste relevant sections from the manuscript (include quotes in quotation marks "like this" to indicate direct quotes from your manuscript), or elaborate on this item by providing additional information not in the ms, or briefly explain why the item is not applicable/relevant for your study

"The authors declare that they have no competing interests related to this study."

About the CONSORT EHEALTH checklist

As a result of using this checklist, did you make changes in your manuscript? *

- ○ yes, major changes
- ◉ yes, minor changes
- ○ no

What were the most important changes you made as a result of using this checklist?

adding informations in the abstract

How much time did you spend on going through the checklist INCLUDING making changes in your manuscript *

5h

As a result of using this checklist, do you think your manuscript has improved? *

- ◉ yes
- ○ no
- ○ Autre :

Would you like to become involved in the CONSORT EHEALTH group?

This would involve for example becoming involved in participating in a workshop and writing an "Explanation and Elaboration" document

- ○ yes
- ◉ no
- ○ Autre :

Effacer la sélection

Any other comments or questions on CONSORT EHEALTH

Votre réponse

STOP - Save this form as PDF before you click submit

To generate a record that you filled in this form, we recommend to generate a PDF of this page (on a Mac, simply select "print" and then select "print as PDF") before you submit it.

When you submit your (revised) paper to JMIR, please upload the PDF as supplementary file.

Don't worry if some text in the textboxes is cut off, as we still have the complete information in our database. Thank you!

Final step: Click submit !

Click submit so we have your answers in our database!

Envoyer Effacer le formulaire

N'envoyez jamais de mots de passe via Google Forms.

Ce contenu n'est ni rédigé, ni cautionné par Google. - Contacter le propriétaire du formulaire - Conditions d'utilisation - Règles de confidentialité

Ce formulaire vous semble suspect ? Signaler

Google Forms
